# Overcoming Chemoimmunotherapy‐Induced Immunosuppression by Assemblable and Depot Forming Immune Modulating Nanosuspension

**DOI:** 10.1002/advs.202102043

**Published:** 2021-08-07

**Authors:** Seung Mo Jin, Sang Nam Lee, Jung Eun Kim, Yeon Jeong Yoo, Chanyoung Song, Hong Sik Shin, Hathaichanok Phuengkham, Chang Hoon Lee, Soong Ho Um, Yong Taik Lim

**Affiliations:** ^1^ SKKU Advanced Institute of Nanotechnology (SAINT) Department of Nano Engineering and School of Chemical Engineering Sungkyunkwan University (SKKU) 2066 Seobu‐ro, Jangan‐gu Suwon Gyeonggi‐do 16419 Republic of Korea

**Keywords:** adjuvants, drug delivery, immunosuppression, immunotherapy, tumor microenvironment

## Abstract

The deficiency of antigen‐specific T cells and the induction of various treatment‐induced immunosuppressions still limits the clinical benefit of cancer immunotherapy. Although the chemo‐immunotherapy adjuvanted with Toll‐like receptor 7/8 agonist (TLR 7/8a) induces immunogenic cell death (ICD) and in situ vaccination effect, indoleamine 2,3‐dioxygenase (IDO) is also significantly increased in the tumor microenvironment (TME) and tumor‐draining lymph node (TDLN), which offsets the activated antitumor immunity. To address the treatment‐induced immunosuppression, an assemblable immune modulating suspension (AIMS) containing ICD inducer (paclitaxel) and supra‐adjuvant (immune booster; R848 as a TLR 7/8a, immunosuppression reliever; epacadostat as an IDO inhibitor) is suggested and shows that it increases cytotoxic T lymphocytes and relieves the IDO‐related immunosuppression (TGF‐*β*, IL‐10, myeloid‐derived suppressor cells, and regulatory T cells) in both TME and TDLN, by the formation of in situ depot in tumor bed as well as by the efficient migration into TDLN. Local administration of AIMS increases T cell infiltration in both local and distant tumors and significantly inhibits the metastasis of tumors to the lung. Reverting treatment‐induced secondary immunosuppression and reshaping “cold tumor” into “hot tumor” by AIMS also increases the response rate of immune checkpoint blockade therapy, which promises a new nanotheranostic strategy in cancer immunotherapy.

## Graphical abstract

Assemblable immune modulating suspension (AIMS) is prepared by immunogenic cell death (ICD) inducer (paclitaxel) and supra‐adjuvant (Toll‐like receptor 7/8a as an immune booster and indoleamine 2,3‐dioxygenase (IDO) inhibitor as an immunosuppression reliever), enabling the relieving of chemo‐immunotherapy‐induced immunosuppression. Reverting treatment‐induced secondary immunosuppression and reshaping “cold tumor” into “hot tumor” by AIMS increases the response rate of immune checkpoint blockade therapy.

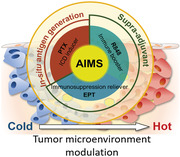

## 1 Introduction

To overcome the low therapeutic efficacy of current immunotherapy such as cancer vaccine, immune checkpoint blockade (ICB), and engineered T cells, various therapeutic modalities including chemotherapy have been combined with them.^[^ ^1^ ^]^ For example, ICB therapy has shown a weak clinical response in patients with malignant tumors, which is attributed to 1) low antigen‐loading, 2) low T cell infiltration, and 3) high expression of immunosuppressive factors; such tumors are characterized as “cold tumors”.^[^ ^2^, ^3^, ^4^ ^]^ Various chemo‐immunotherapeutic modalities including anticancer agents and small molecule‐based immunomodulatory drugs that can address the weak clinical responses were considered as candidates for the combination with ICB.^[^ ^5^, ^6^ ^]^ Toll‐like receptors (TLRs) are the primary receptors for pathogen‐associated molecule patterns (PAMPs) that play a crucial role in inducing innate immunity and orchestrate the subsequent adaptive immune responses against specific tumors.^[^ ^7^, ^8^ ^]^ Various vaccine adjuvants, including TLR agonists, have been developed to synergize ICB‐based immunotherapy by enhancing the immunogenicity of tumor antigens, increasing antigen‐specific T cell lymphocytes, and modulating immunosupprssive cells.^[^ ^9^, ^10^ ^]^ However, the effectiveness of the adjuvant‐induced immune response also elicits a negative feedback mechanism, called indoleamine 2,3‐dioxygenase (IDO), which offsets the activated antitumor immunity and promotes immune evasion by tumors. Indeed, the expression of IDO was significantly increased in the tumor microenvironment (TME) and tumor‐draining lymph node (TDLN) after treatment with TLR 7/8 agonists (TLR 7/8a), suggesting that IDO was induced to counterbalance the increased inflammatory conditions. IDO is an inducible enzyme that is overexpressed in many cells, including tumor and antigen‐presenting cells (APCs), in response to diverse pro‐inflammatory signals.^[^ ^11^, ^12^, ^13^ ^]^ IDO catalyzes the conversion of tryptophan, an essential amino acid required for cell proliferation, to its toxic metabolite kynurenine, resulting in the anergy of effector T cells and an increase in regulatory T cells and myeloid‐derived suppressor cells (MDSCs) in both TME and TDLN, following the formation of an immunosuppressive microenvironment.^[^ ^14^, ^15^, ^16^, ^17^ ^]^ Therefore, the control of IDO‐related secondary immunosuppression generated as a negative feedback mechanism after chemo‐immunotherapy as well as intrinsic tumor‐induced immunosuppression is very important.

**Figure 1:**
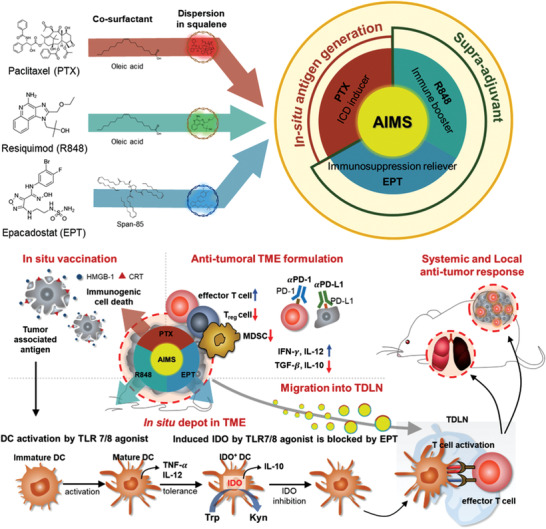
Schematic illustration of an assemblable immune modulating suspension (AIMS)‐based boosting of T cell activity, overcoming the indoleamine 2,3‐dioxygenase (IDO)‐related immunosuppressive tumor microenvironment (TME), and revoking a systemic immune response for the treatment of metastatic tumors. By the formation of in situ depot in tumor bed as well as by the efficient migration into tumor draining lymph node (TDLN), AIMS loaded with supra‐adjuvant [R848 (TLR 7/8 agonist, immune booster), epacadostat (EPT) (IDO inhibitor, immunosuppression reliever)], and paclitaxel (PTX) (immunogenic cell death (ICD) inducer) in a single injection formulation increases immune recognition of tumor antigen and downregulates immunosuppressive factors and cells in the TME, resulting in an enhanced immune checkpoint blockade response.

To address the limitation, we suggest a nanosuspension‐based immune modulation strategy that can induce high population of antigen‐specific T cells and relieve the IDO‐related immunosuppression in both TME and TDLN, through an assemblable immune modulating suspension (AIMS) that can not only form in situ depot in tumor bed but also migrate efficiently into TDLN. We designed and synthesized AIMS containing three components separately; 1) ICD inducer (paclitaxel (PTX), chemotherapeutic agent), 2) immune booster (R848, TLR 7/8a), and 3) immunosuppression reliever (epacadostat (EPT), IDO inhibitor). The incorporation of PTX generates tumor antigens in vivo and induces in situ vaccination, which exploits all relevant antigens in the tumor without the identification of tumor antigens, as an entire array of mutated epitopes is included.^[^ ^18^, ^19^ ^]^ The synergistic combination of R848 as TLR 7/8a and EPT as an IDO inhibitor, termed as a supra‐adjuvant, can generate a synergistic effect that involves the up‐regulation of immune‐promoting factors based on tumor antigens and the simultaneous down‐regulation of immunosuppressive factors. These three components can be lyophilized for long term storage at room temperature, easily reconstituted, and assembled at the desired ratio in the form of a single injection by simple mixing before injection. Local treatment with AIMS can not only reprogram the TME and TDLN, but also elicit systemic immunity, resulting in the prevention of tumor recurrence and metastasis (**Figure** **1**).

## 2 Results

**Figure 2:**
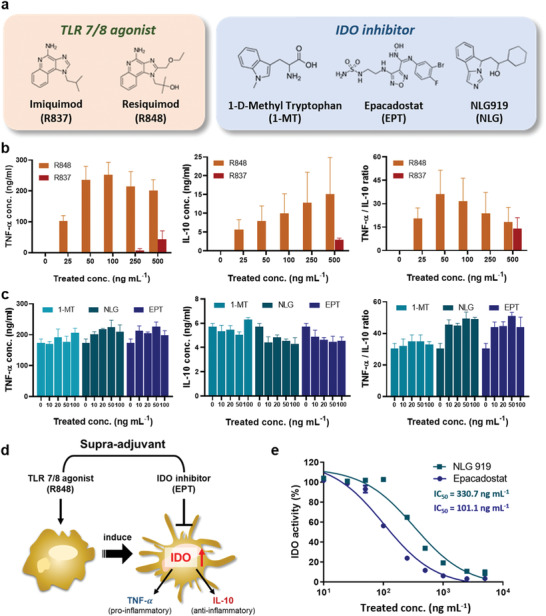
Screening the candidates for synergistic combination as a supra‐adjuvant. a) Representative examples of toll‐like receptor 7/8 agonists (TLR7/8a) and IDO inhibitors. b,c) The effect of TLR 7/8a and IDO inhibitors in antigen‐presenting cells is characterized by the release of cytokines. Levels of TNF‐*α* (proinflammatory), IL‐10 (anti‐inflammatory), and their ratio (TNF‐*α*/IL‐10) were evaluated via ELISA, following treatment with b) TLR 7/8a alone (*n* = 6) or c) 50 ng mL^−1^ R848, in combination with IDO inhibitor (*n* = 3) in RAW 264.7 cells. d) Schematic illustration of the mechanism induced by supra‐adjuvant (R848 and EPT). e) Concentration‐dependent IDO inhibition was compared between NLG 919 and EPT.

A supra‐adjuvant is composed of a synthetic TLR 7/8a and an IDO inhibitor, and representative examples of those have been listed (TLR 7/8a: R837 and R848; IDO inhibitors: 1‐methyl tryptophan (1‐MT), NLG919, and EPT) in **Figure** **2**a. TLR 7/8a—stimulators of TLR 7/8 on APCs—are involved in APC maturation and increased release of proinflammatory cytokines.^[^ ^20^, ^21^ ^]^ However, these proinflammatory signals will subsequently also induce the expression of IDO in APCs, which also increases the release of anti‐inflammatory cytokines (Figure 2b). Therefore, there will be a synergistic effect of the combination of a TLR 7/8a and an IDO inhibitor, and its impact will be observed as an increase in the ratio of pro‐ and anti‐inflammatory cytokines compared to that in cells treated with the TLR 7/8a alone. TNF‐*α* and IL‐10 were used as representative pro‐ and anti‐inflammatory cytokines, respectively, and their secretion levels were quantified via ELISA, following the treatment of RAW 264.7 cells after 24 h of incubation. First, a comparison between R837 and R848—as immune boosters—was made at various concentrations, and R848 (50 ng mL^−1^) was found to be the strongest immune booster in RAW 264.7 cells (Figure 2c). Combination with an IDO inhibitor at various concentrations not only increased the level of TNF‐*α*, but also decreased the level of IL‐10, compared to those of the TLR 7/8a only treatment, which proves the role of the IDO inhibitor as an immunosuppression reliever (Figure 2d). Combination with NLG919 or EPT increased the TNF‐ *α*/IL‐10 ratio more dramatically than combination with 1‐MT. Furthermore, based on the half maximal inhibitory concentration (IC_50_) of the IDO activity in HeLa cells (HeLa assay), EPT was found to have a comparative advantage over NLG919 as an IDO inhibitor (Figure 2e). Therefore, we conducted the subsequent experiments using the chosen combination, R848 and EPT.

**Figure 3:**
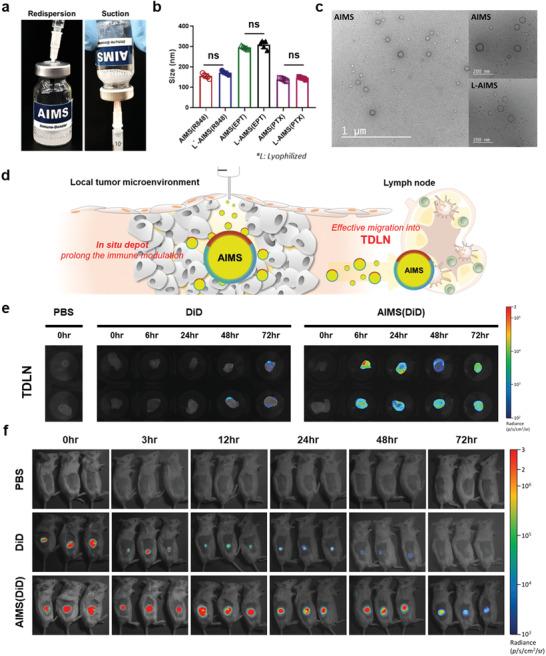
AIMS platform provides favorable characteristics for antitumor immune response: lyophilizable formulation, in situ depot formation, and migration into TDLN. a) Redispersion and suction of lyophilized AIMS by 21 G syringe. b) Mean size of various AIMS containing each drug (*n* = 5). c) TEM image of AIMS platform and lyophilized AIMS (scale bar = 200 nm). d) Drug delivery characteristics: in situ depot formation in TME and efficient migration into TDLN. Fluorescence images depicting e) the lymph node of mice separated 0, 6, 24, 48, and 72 h after injection and f) retention of DiD around the injection site 0, 3, 12, 24, 48, and 72 h after injection. The mice were treated with 50 µL of PBS, 10 mg mL^−1^ DiD dye‐containing solution, or the same concentration of DiD‐containing AIMS intratumorally 5 d after tumor inoculation. Data are presented as the mean ± SD. *P* values were determined by Student's *t* test at the endpoint (ns, not significant).

**Figure 4:**
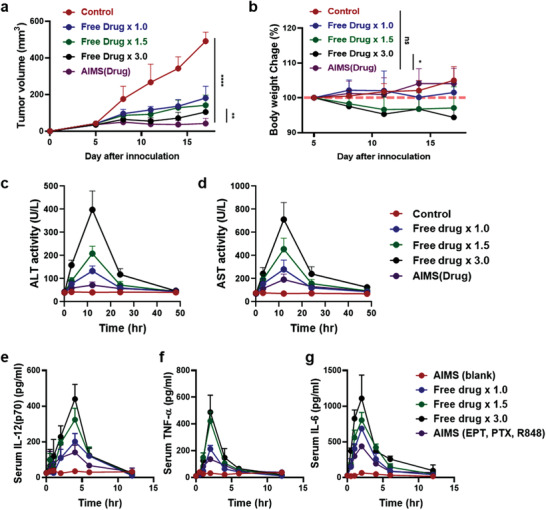
AIMS platform reduces in vivo toxicity of drugs with increased anti‐tumor efficacy. a) tumor growth curve and b) body weight curve of the mice which were treated with PBS (control), 1.5‐ and 3.0‐times higher amounts of drug solubilized in PBS, and drug‐containing AIMS (*n* = 5). c) ALT activity, d) AST activity, serum e) IL‐12(p70), f) TNF‐*α*, and g) IL‐6 cytokine level is measured with same groups (*n* = 3). Drug means the combination of 25 µg of R848, 25 µg of EPT, and 25 µg of PTX. Each sample was treated four times, 5 d after tumor inoculation at 3 d intervals, and the tumor growth and body weight were calculated. Data are presented as the mean ± SD. *P* values were determined by Student's *t* test at the endpoint (*P < 0.05, **P < 0.01, ****P < 0.001; ns, not significant).

Oil‐in‐water (O/W) nano‐emulsion adjuvants have been widely studied and are known to effectively induce immunogenicity; recruit immune cells, including monocytes/macrophages and dendritic cells, to the local environment; and elicit innate and subsequent adaptive immune responses.^[^ ^22^, ^23^ ^]^ Nano‐emulsion‐based adjuvants such as MF59, AS01, and AF03 are licensed as influenza vaccines and have been shown to be safe for human use, according to large safety databases.^[^ ^24^ ^]^ Although they can be used as candidates for enhancing the humoral immune response, they still have a drawback of inducing the cellular immune response.^[^ ^25^ ^]^ Adjuvant immunogenicity can be strongly enhanced or modified by combination with other immunostimulants and their formulations.^[^ ^26^ ^]^ Several small molecule‐based TLR 7/8a are attractive immunostimulants for inducing cellular immune responses and enhancing the efficacy of cancer immunotherapy.^[^ ^27^, ^28^ ^]^ Nano‐emulsions are attractive materials as base adjuvants since they possess a large area in the oil phase where water‐insoluble small molecules can be positioned. Therefore, considering the intrinsic immunogenicity of nano‐emulsions, we decided to load a supra‐adjuvant along with an ICD inducer to maximize their therapeutic effect. AIMS is a squalene‐based O/W nano‐emulsion. We successfully loaded various kinds of small molecules in squalene oil with the aid of oleic acid, Span 85, and Tween 80 and generated a library of synthesizable AIMS systems (Table S1 and Figure S1, Supporting Information). The small molecules are representative examples of immune boosters, immunosuppression relievers, and ICD inducers; each composition can be separately and stably formulated in AIMS. It should be emphasized that AIMS, loaded with each component, is lyophilizable and can be stably stored at room temperature for a long period of time (**Figure** **3**a,b; Figure S2, Supporting Information). Immediate use, by simple reconstitution is also possible, and they can be assembled for a single injection, at a desired ratio, merely by mixing. The AIMS stably maintained its morphology after the reconstitution confirmed by transmission electron microscopy (TEM) image (Figure 3c). As shown in Figure 3b,c, the size of AIMS—measured by dynamic light scattering (DLS) and TEM —was found to be sub‐200 nm (DLS: 150–190 nm; TEM: around 100 nm), and the direct diffusion of AIMS, into the lymph nodes, through lymphatic vessels is highly expected.^[^ ^29^ ^]^ Indeed, intratumorally injected—AIMS labelled with hydrophobic dye, DiD (as a model for hydrophobic drugs) —immediately migrated to the TDLN (≈6 h), whereas the free DiD dye showed a weak signal in the TDLN (Figure 3d,e). The IDO inhibitor also plays an important role in the TDLN, as IDO induces anergy of effector T cells and shifts CD4^+^ T cells to Treg cells during T‐cell priming.^[^ ^30^, ^31^ ^]^ Therefore, the direct migration capacity of AIMS into TDLN is crucial. With the help of AIMS, IDO inhibitors can block IDO activity during T‐cell priming in the TDLN. Moreover, intratumorally injected AIMS(DiD) showed high fluorescence intensity in the tumor for 2–3 d, whereas free DiD started to lose its fluorescence 3 h after injection (Figure 3f). The longer retention of AIMS in tumors is believed to be caused by the interaction between the suspension droplets and the tissue. Due to these properties, AIMS loaded with small molecule‐based immune modulators showed superior antitumor efficacy compared to that of small molecules in the free drug formulation (**Figure** **4**a). This phenomenon implies that the AIMS formulation increased the antitumor activity of small molecule‐based drugs. As we have shown, even threefold higher amounts of the free drug showed lower antitumor activity than the AIMS formulation. However, large amounts of the free drug resulted in significant body weight loss, while mice immunized with the AIMS formulation did not show significant loss in body weight (Figure 4b). Major organs including lung, kidney, and liver were collected and sliced for H&E‐staining. The staining indicated that cell distortion in kidney and liver were detected in threefold higher amounts of the free drug injected group (circled points), while no noticeable signs of organ damage appeared in AIMS formulation immunized group (Figure S3, Supporting Information). Moreover, all the free drug immunized groups showed a higher level of alanine aminotransferase (ALT), aspartate aminotransferase (AST), and cytokines (IL‐12p70, TNF‐*α*, and IL‐6) in serum than AIMS formulation immunized group (Figure 4c,d,e,f,g). The serum level of ALT and AST activity of AIMS formulation immunized group were in normal ranges (normal range in BALB/c mice; ALT: 15–84 U per L, AST: 54–298 U per L).^[^ ^32^, ^33^ ^]^ All these results imply that the AIMS reduced the systemic toxicity.

**Figure 5:**
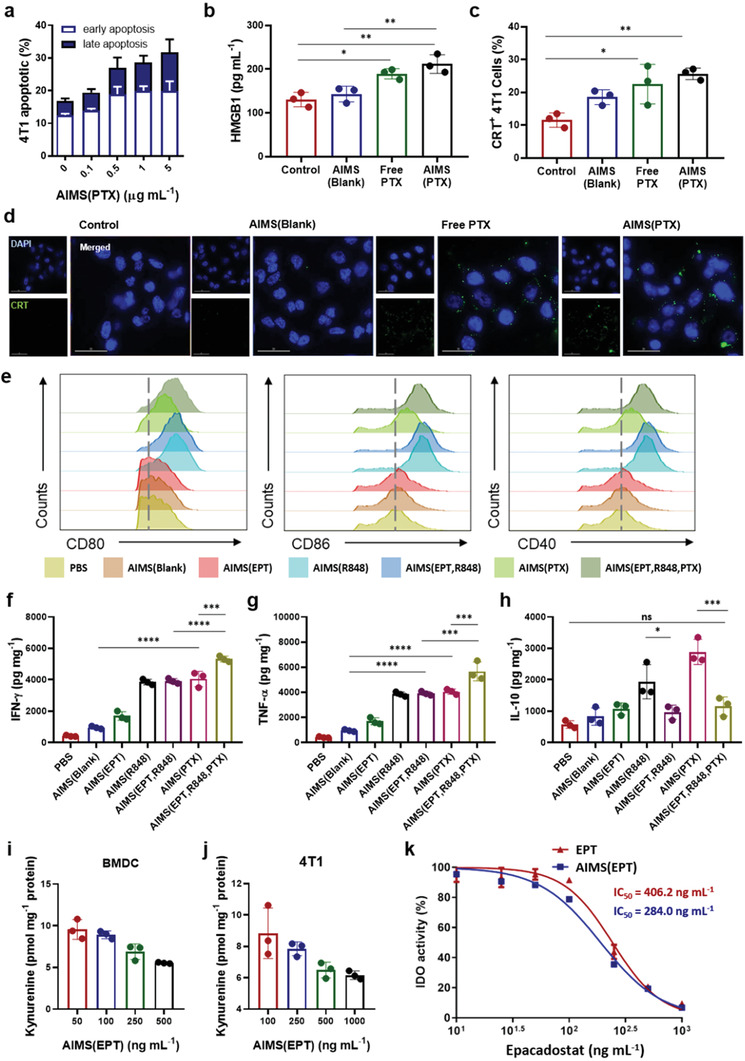
In vitro test of ICD induced by paclitaxel and immune modulation by the supra‐adjuvant. a) Early and late apoptosis induced by AIMS(PTX) depends on concentration (*n* = 3). AIMS(PTX)‐induced ICD characterized by b) the release of HMGB1 detected by ELISA in 4T1 cell culture supernatants (*n* = 3), c) calreticulin (CRT) exposure confirmed by flow cytometry (*n* = 3), and d) Fluorescence image of CRT expression in the 4T1 cell line (scale bar = 30 mm). e) Flow cytometry analysis of the expression of costimulatory markers in BMDCs (CD11c^+^ gated) after treatment. Quantification of f) IFN‐*γ*, g) TNF‐*α*, and h) IL‐10 produced by BMDCs, following treatment with PBS, AIMS(Blank), AIMS(EPT), AIMS(R848), AIMS(EPT, R848), or AIMS(PTX)‐treated 4T1 cell culture medium and their combination (*n* = 3). i,j) The ability of AIMS(EPT) to inhibit IDO activity in both activated i) BMDCs and j) 4T1 tumor cells was assessed by the IDO activity assay (*n* = 3). k) Comparison of IDO inhibitory ability between free EPT and AIMS(EPT) assessed with a HeLa cell‐based IDO assay (*n* = 3). Data are presented as the mean ± SD. *P* values were determined by one‐way ANOVA (*P < 0.05, **P < 0.01, ***P < 0.005, ****P < 0.001; ns, not significant).

PTX is one of the first‐line chemotherapeutic agents used in the clinic that leads to direct tumor cell death.^[^ ^34^ ^]^ Its direct tumor‐killing ability was confirmed by the apoptosis of 4T1 tumor cells; 5 µg mL^−1^ AIMS(PTX) induced apoptosis, at a frequency 2.01‐fold higher than that of AIMS(blank) (**Figure** **5**a). Although the apoptosis of 4T1 tumor cells was strongly induced by AIMS(PTX), this formulation did not exhibit any significant toxicity toward bone marrow‐derived dendritic cells (BMDCs) at similar concentrations (Figure S4, Supporting Information). Surprisingly, proliferation of BMDCs was observed, demonstrating that BMDCs are resistant to the toxicity of AIMS(PTX).^[^ ^35^, ^36^ ^]^ PTX is known to act as a conventional chemotherapeutic agent and as an activator of the immune responses via ICD induction. ICD is characterized by the extracellular secretion of high mobility group box 1 (HMGB1, a danger signal) and, exposure of calreticulin (CRT, eat‐me signal) on the cell surface which are classified as danger‐associated molecular patterns (DAMPs).^[^ ^37^, ^38^, ^39^ ^]^ AIMS(PTX) treatment of 4T1 tumor cells induced extracellular release of HMGB1 which is characterized by ELISA (Figure 5b). AIMS(PTX) also successfully induced CRT expression on the 4T1 tumor cell surface, as observed by fluorescence microscopy and quantified by flow cytometry (Figure 5c,d). DAMPs generated by AIMS(PTX)‐induced ICD can elicit a strong immune response.^[^ ^38^ ^]^ AIMS(PTX) —a potent ICD inducer—generates not only DAMPs but also tumor‐associated antigens that function as antigen sources in cancer vaccines. Therefore, a supra‐adjuvant combined with ICD‐inducible AIMS(PTX) can elicit in situ vaccination, which maximizes the anti‐tumoral immune response of the supra‐adjuvant.

To evaluate the immunomodulatory effect of immune modulators (EPT, R848, and PTX) in AIMS formulation, we investigated their impact on the maturation of DCs. Mature DCs—which play a pivotal role in antigen‐specific T cell immunity^[^ ^40^ ^]^—present two significant signals that can determine the fate of naïve T cells.^[^ ^11^, ^41^ ^]^ These are the co‐stimulatory markers (CD40, CD80, and CD86)—expressed on the surface of DCs—and the secreted cytokines. BMDCs isolated from BALB/c mice were incubated with AIMS(blank), AIMS(EPT), AIMS(R848) and AIMS(PTX)‐treated 4T1 tumor cell medium. AIMS(R848) successfully up‐regulated co‐stimulatory marker expression relative to that of AIMS(blank), which suggests that the TLR 7/8a increased the immunogenicity of AIMS(blank). AIMS(PTX) treated‐4T1 culture medium, which consists of DAMPs (HMGB1 and CRT) —generated by the ICD of 4T1—also significantly increased co‐stimulatory marker expression (Figure 5e). Compared with AIMS(R848) and AIMS(PTX), AIMS(EPT) did not induce the upregulation of co‐stimulatory markers. AIMS(blank), AIMS(R848), and AIMS(PTX)‐treated 4T1 tumor cell media increased the release of proinflammatory cytokines (IL‐12(p70) and TNF‐*α*) (Figure 5f,g). However, the levels of an anti‐inflammatory cytokine (IL‐10) were also increased, which could be attributed to IDO expression induced by the increased proinflammatory signals (Figure 5h). Thus, activated IDO turned out to modulate the APCs to produce immune‐suppressive cytokines (anti‐inflammatory cytokines), such as IL‐10, which consequently blocked the production of proinflammatory cytokines, such as IL‐12(p70) and TNF‐*α*. Interestingly, combination with AIMS(EPT), which blocks IDO activity, not only reduced the release of anti‐inflammatory cytokines dramatically but also increased the secretion of proinflammatory cytokines, suggesting that AIMS(EPT) supported the reprogramming of APCs. Inhibition of IDO activity by AIMS(EPT), in both mature DCs and 4T1 tumor cells, was assessed by an IDO activity assay. To mimic the IDO gene expression induced by proinflammatory signals, BMDCs were pre‐treated with 200 ng mL^−1^ LPS, and 4T1 tumor cells were pre‐treated with 100 ng mL^−1^ IFN‐*γ*. Treatment with AIMS(EPT) at the desired concentration was performed, and the cells were incubated for 24 h. Inhibition of IDO activity was then assessed by measuring the amount of kynurenine converted from tryptophan; AIMS(EPT) successfully reduced kynurenine formation in both BMDCs (Figure 5i) and 4T1 tumor cells (Figure 5j). A comparison of IDO inhibition ability between free EPT and AIMS(EPT) was performed using a standard HeLa cell‐based IDO assay (Figure 5k). Free EPT inhibited 50% of the IDO activity (IC_50_) at 242.0 ng mL^−1^, whereas AIMS(EPT) had an IC_50_ of 190.2 ng mL^−1^, which was 1.27‐fold lower than that of free EPT. AIMS(EPT) increased IDO inhibition ability compared with that of free EPT, probably due to the superior delivery of EPT into the cell by AIMS.

**Figure 6:**
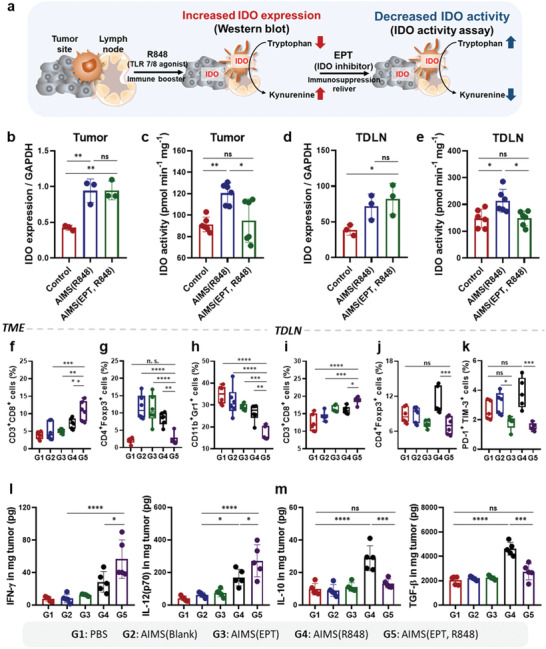
Supra‐adjuvant induced the modulation of immune profile in the TME and TDLN. a) Schematic depiction of IDO expression induced by treatment with AIMS(R848) and subsequent blocking of IDO activity by AIMS(EPT). Mice were treated four times, at 3 d intervals, when the tumors reached 50–60 mm^3^ in size. Western blot analysis of IDO‐1 expression are quantitatively analyzed with normalization of GAPDH in b) TME and c) TDLN (*n* = 3). IDO activity in d) TME and e) TDLN was measured by an IDO activity assay (*n* = 6). Flow cytometry analysis showing the populations of f) CD3^+^CD8^+^ T cells, g) CD4^+^Foxp3^+^ Treg cells, and h) CD11b^+^Gr‐1^+^ MDSCs in the TME (*n* = 6). Flow cytometry analysis showing the populations of i) CD3^+^CD8^+^ T cells, j) CD4^+^Foxp3^+^ Treg cells, and k) PD1^+^TIM‐3^+^ exhausted T cells in TDLN (*n* = 6). l) Concentrations of proinflammatory cytokines (IFN‐*γ* and IL‐12(p70)) in the TME and TDLN (*n* = 5). m) Concentrations of anti‐inflammatory cytokines (IL‐10 and TGF‐*β*) in the TME and TDLN (*n* = 5). Data are presented as the mean ± SD. *P* values were determined by one‐way ANOVA (*P < 0.05, **P < 0.01, ***P < 0.005, ****P < 0.001; ns, not significant).

As demonstrated, the potent immunogenicity of TLR 7/8a adversely affected the levels of immune‐suppressive factors such as IDO and generated an immune‐suppressive TME (**Figure** **6**a). Therefore, cotreatment of TLR 7/8a with an IDO inhibitor, prevented the formation of an immune‐suppressive environment and maximized the effectiveness of these agonists as adjuvants. To evaluate the synergistic effect of EPT and R848, we analyzed the modulated infiltrated immune cell population in the TME and TDLN of tumor‐bearing mice. Murine 4T1 triple‐negative breast tumors were grown to a size of 50–60 mm^3^ (5 d after 5 × 10^5^ 4T1 tumor cell injection) on the flank. Then, the mice were intratumorally administered PBS, AIMS(blank), AIMS(R848), AIMS(EPT), or a mix of AIMS(R848) and AIMS(EPT) at the indicated doses four times at 3 d intervals. The mice were euthanized for immune profile analysis 3 d after the last injection. Western blot analysis confirmed that administration of the strong immune booster, R848, induced IDO expression in the TME (Figure 6b; Figure S5a, Supporting information) and TDLN (Figure 6d; Figure S5b, Supporting information). This increase strongly supports the importance of the combination of R848 and an IDO inhibitor, as a supra‐adjuvant. Interestingly, the AIMS(EPT, R848)‐treated group did not exhibit downregulated IDO expression. This could be because the IDO inhibitor did not downregulate the expression of IDO but blocked IDO activity. Therefore, we investigated IDO activity in each group by an IDO activity assay. The AIMS(EPT, R848) treatment successfully lowered IDO activity, which was increased in the AIMS(R848)‐alone treated group, suggesting that AIMS(EPT) is very effective at lowering the induced IDO activity in both the TME (Figure 6c) and the TDLN (Figure 6e).

The trend of tumor growth and tumor weight indicates the superior antitumor efficiency of the supra‐adjuvant with respect to AIMS(R848) (Figure S6, Supporting Information). To understand the mechanism governing the antitumor efficiency of the supra‐adjuvant, we examined the population of infiltrated immune cells in both the TME and in the TDLN with following gating strategy (Figure S7, Supporting Information). Flow cytometry analysis indicated that both AIMS(R848) and AIMS(EPT, R848) were effective in inducing DC maturation (CD80^+^CD86^+^) (Figure S8, Supporting Information). Compared with AIMS(R848)‐only treated mice, mice treated with the AIMS(EPT, R848) exhibited an increase in the effector T cell population in the TME and in the TDLN, in addition to a decrease in CD4^+^FOXP3^+^ regulatory T cell (T_reg_ cell) counts (Figure 6f,g,i,j; Figures S9 and S10, Supporting Information). The AIMS(EPT, R848) treatment also increased the ratios of both CD4^+^ T cells/T_reg_ cells and CD8^+^ T cells/T_reg_ cells in the TME and in the TDLN (Figure S11, Supporting Information). AIMS(EPT, R848) was effective in generating high population of effector memory CD4^+^ or CD8^+^ T cells compared to AIMS(R848) in TDLN (Figure S12, Supporting Information). Interestingly, in TDLN, AIMS(R848) treatment induced the exhaustion of CD8^+^ T cells (PD‐1^+^ TIM‐3^+^), while AIMS(EPT, R848) treatment blocked the exhaustion of CD8^+^ T cells (Figure 6k; Figure S13, Supporting Information). Moreover, MDSCs, a major suppressive cell type in the TME, were also significantly decreased by the AIMS(EPT, R848) (Figure 6h; Figure S14, Supporting Information). The AIMS(R848)‐treated group, and the AIMS(EPT, R848)‐treated group showed increased intratumoral concentrations of proinflammatory cytokines (IFN‐*γ* and IL‐12(p70)), indicating the successful priming of CD8^+^ T cells (Figure 6l). However, treatment with AIMS(R848) increased the local concentration of anti‐inflammatory cytokines (IL‐10 and TGF‐*β*), which was due to IDO‐expressing DCs and macrophages (Figure 6m). As anti‐inflammatory cytokines play a significant role in suppressing the anti‐tumor efficacy in the TME by suppressing the T‐cell‐mediated immunosurveillance,^[^ ^42^ ^]^ the increased level of anti‐inflammatory cytokines may neutralize T cell immunity. Interestingly, AIMS(EPT, R848) treatment down‐regulated the increased in secretion of anti‐inflammatory cytokines so that it reached the level observed in the control group. All these findings demonstrate that the supra‐adjuvant clearly down‐regulated the immune‐suppressive factors induced by AIMS(R848) and generated an antitumoral environment.

As mentioned above, supra‐adjuvant combined with ICD‐inducible AIMS(PTX) can elicit in situ vaccination, which maximizes the anti‐tumoral immune response of the supra‐adjuvant. The effective dose of AIMS(EPT, R848, PTX) was determined as below. In Figure 2, we have conducted the in vitro screening of candidates for supra‐adjuvant, based on the value of TNF‐*α*/IL‐10 through serial dilution of each drug, and the most effective ratio of the EPT and R848 combination was 1:1 ratio (50 ng mL^−1^ of R848 and 50 ng mL^−1^ of EPT). This 1:1 ratio was kept in further in vivo experiments (25 µg per mice of R848 and 25 µg per mice of EPT). Then, to determine the suitable dose of AIMS(PTX) for the combination with AIMS(EPT, R848), AIMS(EPT, R848) was co‐treated with various concentration of AIMS(PTX). Then we examined the body weight change for toxicity index and tumor growth curve for therapeutic index (Figure S15, Supporting Information). Supra‐adjuvant combined with 25 µg of AIMS(PTX) showed considerable anti‐tumor effects compared to 50 µg of AIMS(PTX) and showed none of the body weight loss compared to 50 or 100 µg of AIMS(PTX). Therefore, we decided to incorporate 25 µg of PTX into the AIMS system for the component of therapeutic cancer vaccine. Next, mice immunized with AIMS(EPT, R848, PTX) exhibited a significantly reduced tumor volume by more than 2.39‐fold and a prolonged survival rate compared with that of mice immunized with AIMS(EPT, R848), indicating the active antitumor activity of AIMS(EPT, R848, PTX) against local tumors (Figure S16, Supporting Information).

**Figure 7:**
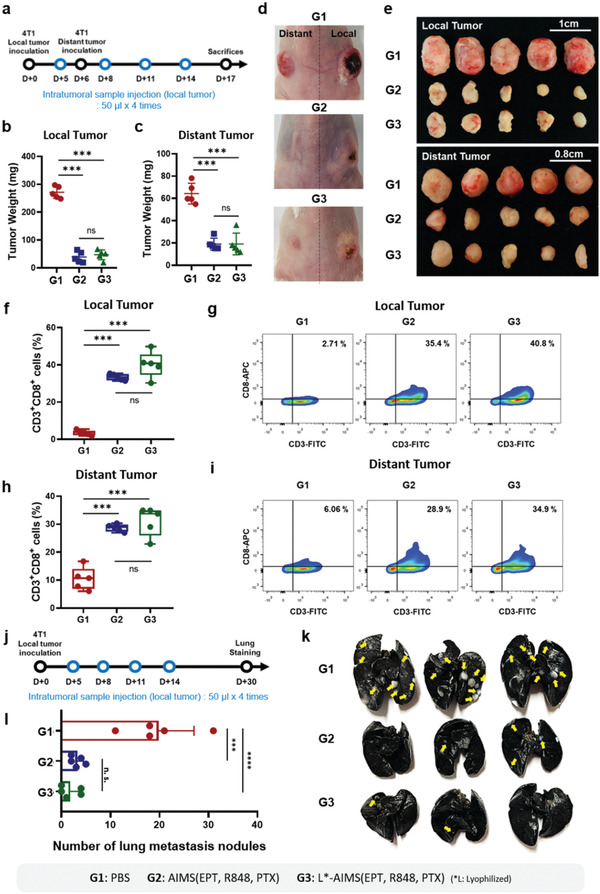
Generation of a systemic antitumor immune response by AIMS and lyophilized AIMS. a) Treatment schedule in the distant 4T1 tumor‐bearing BALB/c mouse model. The tumor weight of b) local and c) distant tumors (*n* = 5). Representative photographs of d) mice, and e) detached tumor images of each group (*n* = 5). f–i) Flow cytometry analysis and corresponding representative data show the population of CD3^+^CD8^+^ T cells in f,g) local and h,i) distant tumors (*n* = 5). j) Treatment schedule for lung metastasis analysis in a 4T1 tumor‐bearing BALB/c mouse model. k) Lung photograph showing metastatic tumors from mice 30 d after tumor inoculation (yellow arrow; tumors in the lung) and i) corresponding quantification of the number of metastatic lung nodules (*n* = 5). Data are presented as the mean ± SD. *P* values were determined by one‐way ANOVA (***P < 0.005, ****P < 0.001; ns, not significant).

To investigate whether local treatment with AIMS(EPT, R848, PTX) induced a systemic antitumor immune response, we analyzed the antitumor effect of AIMS(EPT, R848, PTX) on distant tumor and lung metastatic tumors using the schedules shown (**Figure** **7**a,j). To analyze the effect on distant tumors, tumor cells were inoculated on the opposite side of local tumors and the treatment was performed only intratumorally on local tumors. AIMS(EPT, R848, PTX) not only inhibited local tumor growth, but also significantly eliminated distant tumor growth 3.38‐fold more efficiently than PBS (Figure 7b,c,d,e). Interestingly, reconstituted, lyophilized AIMS(EPT, R848, PTX) also exhibited almost identical antitumoral immunity against distant tumors. The systemic antitumor effect observed against both local tumor (Figure 7f,g) and distant tumors can be attributed to the increased tumor infiltration of CD8^+^ T cells (Figure 7h,i). Second, spontaneous metastasis of the tumor into the lung was examined. Although, a number of metastatic tumor nodules in the lung were detected in the naïve group, there were no noticeable signs of metastasis in the AIMS(EPT, R848, PTX)‐ or Lyophilized‐AIMS (L‐AIMS) (EPT, R848, PTX)‐treated groups (Figure 7j,k,l). Thus, local treatment with AIMS(EPT, R848, PTX) induced the priming of CD8^+^ T cells in the LN followed by the systemic inhibition of distant tumors and the metastasis of tumors into the lung.

**Figure 8:**
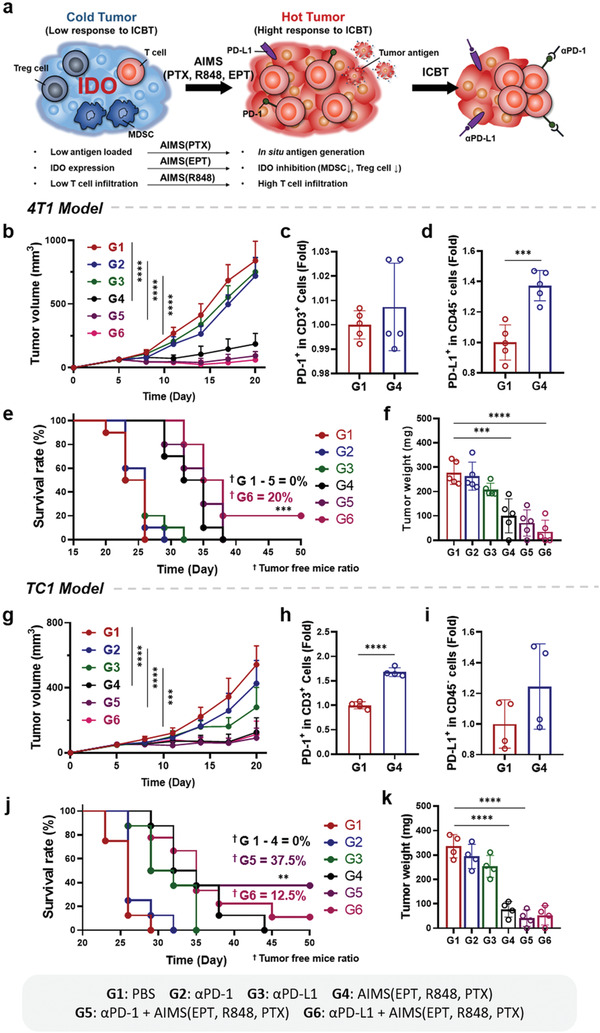
AIMS increased the therapeutic response rate of ICB in 4T1 (b–g) and TC1 (h–m) tumor models. a) Schematic depiction of AIMS reprogramming immunosuppressive TME from “cold tumor” to “hot tumor”, resulting in a stronger therapeutic response to ICB and enhanced antitumor immune activity. Mice were treated as groups noted in figure. Tumor growth curves (*n* = 7) of b) 4T1 and g) TC1 models. All analyses were performed on day 17 after tumor inoculation. Five and four mice were sacrificed for analysis in the 4T1 and TC1 models, respectively. Flow cytometric analysis of PD‐1 expression in CD3^+^ T cells in c) 4T1 and h) TC1 models. PD‐L1 expression in CD45^−^ cells in d) 4T1 and i) TC1 models. Survival curves of e) 4T1‐bearing mice (*n* = 8) and j) TC1‐bearing mice (*n* = 7). Tumor weight of f) 4T1‐bearing mice (*n* = 5) and k) TC1‐bearing mice (*n* = 4). Data are presented as the mean ± SD. *P* values were determined by Student's *t* test, log‐rank test, and one‐way ANOVA (**P < 0.01, ***P < 0.005, ****P < 0.001; ns, not significant).

Despite the emergence of therapeutic monoclonal antibodies targeting immune checkpoints, a lack of response to malignant tumors has been observed.^[^ ^19^ ^]^ To overcome immune resistance, it is necessary to transform an immunologically unresponsive “cold” tumor into a “hot” tumor (**Figure** **8**a).^[^ ^43^ ^]^ “Cold” tumors are characterized by, 1) low antigen loadings, 2) low T cell infiltration, and 3) high expression of immune‐suppressive factors.^[^ ^44^ ^]^ ICD by AIMS(PTX) successfully generated tumor‐associated antigens, and supra‐adjuvant therapy increased CD8^+^ T cell counts in the TME. In addition, the blockade of IDO activity by AIMS(EPT) resulted in the depletion of immune‐suppressive cells (MDSCs and regulatory T cells) and inhibition of immune‐suppressive cytokines (TGF‐*β* and IL‐10) secretion. In this regard, AIMS(EPT, R848, PTX) is expected to increase the response rate of ICB by transforming “cold” tumors into “hot” tumors. Indeed, compared with single treatment with anti‐PD‐1 (*α*PD‐1) or anti‐PD‐L1 (*α*PD‐L1) alone, the combination of *α*PD‐1 or *α*PD‐L1 with AIMS(EPT, R848, PTX) significantly increased the antitumor effect of ICB and increased the survival rate in two different tumor models (4T1 and TC1) (4T1; Figure 8b,e,f, TC1; Figure 8g,j,k). The synergistic effect of AIMS(EPT, R848, PTX) and ICB was verified by the increase in infiltrating CD8^+^ T cell counts in the TME (4T1; Figure S17a, Supporting Information, TC1; Figure S17b, Supporting Information). Interestingly, in the 4T1 tumor model, AIMS(EPT, R848, PTX) treatment increased PD‐L1 expression in CD45^−^ cells, whereas there was no significant increase in PD‐1 expression in CD3^+^ cells (Figure 8c,d; Figure S18a, Supporting Information). The antitumor effect (tumor free mice ratio was 20%) and the increase in infiltrated CD8^+^ T cells were also greater when AIMS(EPT, R848, PTX) was combined with *α*PD‐L1 than when it was combined with *α*PD‐1, suggesting that blocking PD‐L1 is better than blocking PD‐1 after AIMS(EPT, R848, PTX) treatment. In contrast, interestingly, in the TC1 tumor model, AIMS(EPT, R848, PTX) treatment increased PD‐1 expression in CD3^+^ cells, whereas there was no statistically significant increase in PD‐L1 expression in CD45^−^ cells (Figure 8h,I; Figure S18b, Supporting Information). The antitumor effect was also greater when AIMS(EPT, R848, PTX) was combined with PD‐1(tumor free mice ratio was 37.5%) than when it was combined with PD‐L1(tumor free mice ratio was 12.5%), suggesting that blocking PD‐1 is better than blocking PD‐L1 after AIMS(EPT, R848, PTX) treatment. Further systematic researches on the different resistance mechanisms to the combination of AIMS and ICBT in 4T1 and TC1 tumors based on various biomarkers in TME would be helpful to uncover the underlying mechanism of the different responses to *α*PD‐1 and *α*PD‐L1.^[^ ^45^ ^]^ Finally, we verified whether AIMS increased the synergistic anti‐cancer efficacy of EPT, R848, PTX drugs, and immune checkpoint inhibitors (Figure S19, Supporting Information).

## 3 Discussion

In this research, we developed a nanosuspension‐based immune modulation strategy that can overcome the limitation of the clinical benefit of current cancer immunotherapy. Current cancer immunotherapies (such as cancer vaccine, immune checkpoint blockade (ICB), and engineered T cells) combine with various therapeutic modalities to overcome their intrinsic limitations.^[^ ^1^ ^]^ However, most of the combination therapies are now facing the treatment‐induced secondary immunosuppression that should be addressed to enhance the final therapeutic efficacies. For example, although most of the researches on tumor‐induced immunosuppression have focused on the use of ICB (PD‐1, PD‐L1 and CTLA‐4, etc.) inhibitors, the therapeutic efficacy of them still remains at ≈5–30% range.^[^ ^46^ ^]^ The low therapeutic efficacy of ICB is related to various antitumor treatment‐induced immunosuppressions in both tumor microenvironment (TME) and tumor‐draining lymph node (TDLN). Therefore, to increase the therapeutic efficacy of current immunotherapy, the control of secondary immunosuppression generated as a negative feedback mechanism after antitumor therapy as well as intrinsic tumor‐induced immunosuppression should be addressed.

Here, we demonstrated that an assemblable immune modulating suspension (AIMS) can not only form in situ depot in tumor bed but also migrate efficiently into tumor draining lymph node, and the AIMS could be used for the induction of antigen‐specific T cells and the immune converting of IDO‐related immunosuppression in both TMEs and TDLNs. We have observed IDO expression was highly induced in the TME and in the TDLNs after TLR 7/8a treatment (Figure 6) and the IDO expression also affected the infiltrated immune cells (MDSCs, CD8^+^ T cells, and Treg cells) and local cytokine levels (proinflammatory: IL‐12 and IFN‐*γ*; anti‐inflammatory: IL‐10 and TGF‐*β*) (Figure 6). Based on these results, we suggest that TLR 7/8a should be used in combination with IDO inhibitors, and we also propose that this treatment could be realized as supra‐adjuvant therapy. The best combination of a TLR 7/8a and an IDO inhibitor for supra‐adjuvant therapy was evaluated by screening their possible combinations, based on the ratio of pro and anti‐inflammatory cytokines; R848 and EPT showed the most potent synergistic effect. After co‐treatment, the IDO inhibitor could successfully lower the IDO activity induced by the TLR 7/8a at the cellular level (DCs and 4T1 tumor cells) (Figure 5) and at the tissue levels (tumor and TDLN) (Figure 6). The lowered IDO activity by supra‐adjuvant resulted in the increased secretion of proinflammatory cytokine (TNF‐*α*), while decreased secretion of anti‐inflammatory cytokines (IL‐10) in APCs, compared with that observed after TLR 7/8a‐only treatment (Figure 5). In vivo experiments also showed that the overall proinflammatory cytokines (IFN‐*γ* and IL‐12(p70)) level was increased while the anti‐inflammatory cytokine (TGF‐*β* and IL‐10) level in the TME was decreased (Figure 6). It is well known that the increase in TGF‐*β* and IL‐10 in the TME is an inevitable consequence of cancer vaccine immunotherapy, and anti‐inflammatory cytokines are among the main obstacles that reduce anti‐tumor immunity by promoting immune‐suppressive environments.^[^ ^42^, ^47^, ^48^ ^]^ Therefore, the fact that supra‐adjuvants showed an ability to reduce the increased anti‐inflammatory cytokines is significant. Furthermore, supra‐adjuvants were also effective in increasing infiltrated effector T cell counts while decreasing the populations of Treg cells and MDSCs (Figure 6). Thus, IDO inhibitors are effective in overcoming the induced immune‐suppressive TME and supra‐adjuvants can maximize the therapeutic effects of TLR 7/8a‐based cancer vaccines. We have organized the cross‐talk and relationships between infiltrated immune cells and the level of cytokines in the local TME and suggested an effective therapeutic approach to transform the TME into an antitumoral immune niche.

The supra‐adjuvant, combined with the ICD inducer, showed a high potential as a therapeutic cancer vaccine. Direct injection of an ICD inducer generated tumor antigens in vivo, and the interplay with supra‐adjuvant maximized the antigen‐specific antitumoral immune response. AIMS(PTX) treatment of the 4T1 tumor cell line successfully induced the exposure of CRT and the release of HMGB1, which showed that ICD was induced effectively (Figure 5). The in situ vaccination with supra‐adjuvant decreased the tumor weight 2.39‐fold compared with that by supra‐adjuvant treatment only. Intratumorally‐injected AIMS(EPT, R848, PTX) were effective at inhibiting tumor recurrence at the local site as well as in the treatment of distant tumor and the prevention of tumor metastasis into the lung (Figure 7). Systemic immunity was possible because primed T cells in the lymph node could be systemically infiltrated in tumor at distal locations. We observed that the number of infiltrated effector T cells was increased in distant tumors (Figure 7).

The effectiveness of an in situ vaccinated cancer vaccine with a supra‐adjuvant was highly increased in AIMS formulations. AIMS(EPT, R848, PTX) required a smaller volume (one‐third) compared with that of free drug formulation, and induced an effective therapeutic effect without systemic toxicity, which was confirmed by change in body weight, ALT, AST activity, and serum cytokine levels (Figure 4). The superiority of AIMS can be attributed to two main factors. First, the incorporation of drugs into AIMS prolonged their retention in the TME where the action of the IDO inhibitor is essential due to the interaction of suspension droplets with the tissue (Figure 3). Second, the relatively small size of AIMS made it possible for the formulation to migrate directly to the TDLNs, where the action of the IDO inhibitor is also essential (Figure 3). The AIMS(EPT, R848, PTX) described in this study has several advantages from the pharmaceutical point of view. First, its lyophilization capacity ensures stability upon long‐term storage of the formulation even at room temperature. Second, the relative doses of each component can be easily adjusted according to personal need; all three components of AIMS(EPT, R848, PTX) (ICD inducer, immune booster, and immunosuppression reliever) can be fabricated separately and lyophilized. Each component can be adjusted with other components in our AIMS library (Table S1, Supporting Information). After all the determinations are performed, they can be used in a single injection simply by mixing. Third, the easy and robust fabrication of AIMS based on FDA‐approved pharmaceutical ingredients (lipid, oil, and small molecule) is attractive for large‐scale manufacturing.

## 4 Conclusion

Finally, we have demonstrated that the low response rate of ICB in malignant tumors can be increased significantly by co‐treatment with AIMS(EPT, R848, PTX). AIMS(EPT, R848, PTX) was effective in shifting a “cold tumor” to a “hot tumor” in that, 1) ICD was induced by AIMS(PTX)‐generated tumor‐associated antigen, 2) AIMS(EPT) eliminated the immune‐suppressive microenvironment by depleting immune‐suppressive cells (MDSCs and Treg cells) and decreasing the local concentrations of immune‐suppressive cytokines (TGF‐*β* and IL‐10), and 3) supra‐adjuvant increased infiltrating effector T cell counts in the TME (Figure 8). We examined the therapeutic efficacy of the combination of AIMS(EPT, R848, PTX) and ICB in two different tumor models (4T1 and TC1 tumor cells) and observed that tumor volume and weight were decreased significantly and CD8^+^ T cells were highly increased in the combination group compared with the ICBT alone group. Taken together, these results showed that the AIMS(EPT, R848, PTX) system reported here has a potential for use as a nanotherapeutic solution for the control of immunosuppression generated as a negative feedback mechanism after chemo‐immunotherapy as well as tumor‐induced intrinsic immunosuppression.

## Conflict of Interest

The authors declare no conflict of interest.

## Supporting information

Supporting Information

## Data Availability

Research data are not shared.
