# Supplementary material for: Overcoming Chemoimmunotherapy‐Induced Immunosuppression by Assemblable and Depot Forming Immune Modulating Nanosuspension
Source: Adv Sci (Weinh). 2021 Aug 7;8(19):2102043. doi: 10.1002/advs.202102043 (PMC8498862; doi:10.1002/advs.202102043)
Supplement: Supplementary file 1 — Supporting Information [file ADVS-8-2102043-s001.pdf]

WILEY-VCH

© 2021 Wiley-VCH GmbH

ADVANCED SCIENCE
Open Access

Supporting Information

for *Adv. Sci.,* DOI: 10.1002/advs.202102043

Overcoming Chemoimmunotherapy-induced Immunosuppression by Assemblable and Depot Forming Immune Modulating Nanosuspension

*Seung Mo Jin, Sang Nam Lee, Jung Eun Kim, Yeon Jeong Yoo, Chanyoung Song, Hong Sik Shin, Hathaichanok Phuengkham, Chang Hoon Lee, Soong Ho Um, and Yong Taik Lim**

Supporting Information

Overcoming Chemoimmunotherapy-induced Immunosuppression by Assemblable and Depot Forming Immune Modulating Nanosuspension

*Seung Mo Jin, Sang Nam Lee, Jung Eun Kim, Yeon Jeong Yoo, Chanyoung Song, Hong Sik Shin, Hathaichanok Phuengkham, Chang Hoon Lee, Soong Ho Um, and Yong Taik Lim**

Methods

Materials

R848, epacadostat (EPT), and paclitaxel (PTX) were purchased from Med Chem Express (NJ, USA). Span-85, Tween-80, and ethyl alcohol were obtained from Merck (Darmstadt, Germany). Oleic acid and chloroform were purchased from Sigma Aldrich. Phosphate-buffered saline (PBS) was purchased from HyClone (GE Healthcare, South Logan, UT, USA), and Dulbecco's phosphate-buffered saline (DPBS) 10x was purchased from Welgene (Gyeongsan, Korea). RPMI medium 1640 and DMEM were purchased from Gibco (Thermo Fisher Scientific, Waltham, MA, USA). Liquid nitrogen gas was supplied by JC Gas (Korea). DiD was obtained from Invitrogen (Thermo Fisher Scientific, Waltham, MA, USA).

Antibodies

Antibody	Clone	Catalogue number	Company
IDO polyclonal antibody		PA5-24598	Invitrogen
HRP donkey anti-rabbit IgG secondary antibody		A16023	Invitrogen
Anti-calreticulin antibody		Ab2907	Abcam
Goat anti-rabbit IgG secondary		Ab150077	Abcam

antibody			
FITC-conjugated anti-CD11c	HL3	553801	BD Pharmingen
APC-conjugated anti-CD40	3/23	558695	BD Pharmingen
PerCPCy5.5-conjugated anti-CD40	3/23	B262641	BioLegend
APC-conjugated anti-CD80	16-10A1	560016	BD Pharmingen
PE-conjugated anti-CD80	16-10A1	553769	BD Pharmingen
APC-conjugated anti-CD86	GL1	558703	BD Pharmingen
FITC-conjugated anti-CD3	17A2	100204	BioLegend
APC-conjugated anti-CD3	145-2C11	100312	BioLegend
APC-conjugated anti-CD8a	53-6.7	100712	BioLegend
PECy7-conjugated anti-CD8a	53-6.7	552877	BD Pharmingen
PE-conjugated anti-CD4	GK1.5	553730	BD Pharmingen
PerCP-conjugated anti-CD4	GK1.5	100432	BioLegend
APC-conjugated anti-CD45	30-F11	103112	BioLegend
FITC-conjugated anti-FOXP3	FJK-16S	4340671	eBioscience
FITC-conjugated anti-CD44	IM7	553133	BD Pharmingen
PE-conjugated anti-CD62L	MEL-14	553151	BD Pharmingen
PE-conjugated anti-TIM-3 (CD366)	B8.2C12	134004	BioLegend
PerCPCy5.5-conjugated anti-LAG-3 (CD223)	C9B7W	125212	BioLegend
APC-conjugated anti-CD11b	M1/70	101212	BioLegend
FITC-conjugated anti-Ly-6G/Ly-6C (Gr-1)	RB6-8C5	108406	BioLegend
PE-conjugated anti-CD279 (PD-1)	RMP1-14	114118	BioLegend
FITC-conjugated anti-CD279 (PD-1)	29F.1A12	135214	BioLegend

PE-conjugated anti-CD274 (PD-L1)	10F.9G2	124308	BioLegend
Anti-PD-1	RMP1-14	BE0146	Bio X Cell
Anti-PD-L1	10F.9G2	BE0101	Bio X Cell

Mice and cells

BALB/c and C57BL/6 mice (female, 6-week-old) were purchased from Orient Bio (Seongnam, Korea) and maintained under pathogen-free conditions. The animal study was reviewed by the Institutional Animal Care and Use Committee (IACUC) of Sungkyunkwan University School of Medicine, which is accredited by the Association for Assessment and Accreditation of Laboratory Animal Care International (AAALAC International) and abides by the Institute of Laboratory Animal Resources (ILAR) guide. Murine 4T1 cells (triple-negative breast cancer) and murine TC1 cells (cervical cancer) (American Type Culture Collection, ATCC) were cultured in complete RPMI 1640 medium, supplemented with 10 % (v/v) heat-inactivated FBS and 1 % (v/v) penicillin/streptomycin, at 37 °C in a humidified 5 % CO_2 incubator. RAW 264.7 cells (macrophage-like cells) (ATCC) were cultured in DMEM, supplemented with 10 % (v/v) heat-inactivated FBS and 1 % (v/v) penicillin/streptomycin, at 37 °C in a humidified 5 % CO_2 incubator.

Fabrication and lyophilization of AIMS

All AIMSs were emulsified with drug-loaded squalene in PBS solution for 1 min by a probe sonicator (VCX 750, Sonics & Materials, Newtown, CT, USA). For R848, 2 mg of R848, 10 mg of oleic acid, 10 μL of Span-85, and 100 μL of squalene were dissolved in 1 mL of chloroform, and the chloroform was completely evaporated in a desiccator, under vacuum. To load PTX in squalene, the same protocol was used; however, 30 mg of oleic acid was added. To generate EPT-loaded squalene, 2 mg EPT was dissolved in 1 mL of ethyl alcohol with 10 μL of Span-85 and 100 μL of squalene; ethyl alcohol was completely removed. The resulting

drug-loaded squalene solution and 10 μL of Tween-80 were added to PBS, at a final volume of 2 mL. Blank AIMS was fabricated by directly emulsifying 100 μL squalene, 10 μL Span-85, and 10 μL Tween-80 in PBS, at a final volume of 2 mL. All lyophilized AIMS formulations were prepared using the same procedure, except that squalene was dispersed in 10 % (w/v) sucrose-PBS solution. After fabrication, they were frozen in liquid nitrogen for 10 min and stabilized at -20 ℃ for 1 hr. The following lyophilization step was conducted in a freeze-dryer (FDU-2100, EYELA, Japan) under 10 Pa, at -80 ℃ for 3 d. The lyophilized AIMS formulations were sealed with an aluminum cap with a vial cramp (BT1390-20, Daemyung Science, Korea) and reconstituted with distilled water before use.

Characterization of AIMS

The number distribution and zeta potential of the AIMS series were analyzed with DLS, using a zeta-potential & Particle size analyzer (ELSZ-2000 series, Otsuka, Osaka, Japan). To characterize the encapsulation efficiency of AIMS, 500 mL of sample was loaded onto a centrifuge filter (Pierce™ Spin Cups, 0.45 mm, Thermo Fisher Scientific) and centrifuged at 6,000 rpm for 20 min. The filtered solution was collected and analyzed with a UV-visible spectrophotometer (UV-1800, SHIMADZU, Kyoto, Japan). The encapsulation amount was calculated as the ratio of the initially dissolved amount of each drug (2 mg).

Fluorescence imaging of tumor retention and lymph node migration of AIMS

For visualization of AIMS, 0.1 mg of DiD and squalene was dissolved in chloroform, and chloroform was completely evaporated to encapsulate DiD in squalene. DiD-labelled AIMS was fabricated in the same manner as described above. To characterize the retention time and lymph node migration of AIMS from tumor sites, BALB/c mice were subcutaneously inoculated with 5×10^5 4T1 tumor cells in the right flank and maintained for 5 d. After the average tumor volume reached 50 mm^3, PBS, DiD, and DiD-labelled AIMS were

intratumorally injected into mice. For tumor retention time analysis, the injection site was exposed to 650 nm filtered light at 500 mW for 0.2 s, and images were acquired using a 680 nm emission filter at 0, 3, 12, 24, 48 and 72 h. Additionally, lymph nodes were excised from mice 0, 6, 24, 48, and 72 h after injection, and analyzed under the same conditions.

AST and ALT activity analyssis

The blood was collected from each mice injected with PBS (Control), AIMS (EPT, PTX, R848) or free drugs depends on concentration. The collected blood were centrifuged with 10,000 xg for 10 minutes to isolate serum. The serums were analyzed with AST or ALT activity analysis kits (Abcam) to characterize AST or ALT activity.

Serum cytokine level analysis

The blood was collected from each mice injected with AIMS (blank), AIMS (EPT, PTX, R848) or free drugs depends on concentration. The collected blood were centrifuged with 10,000 xg for 10 minutes to isolate serum. The serums were analyzed with ELISA kits (BD Bioscience) to quantify IL-12(p70), TNF-α, and IL-6 concentration.

H&E statining

The organs (Lung, kidney and liver) was isolated from the mice after 4 times injection with 3 days interval of AIMS (EPT, PTX, R848) or free drugs depends on concentration. The isolated organs were sliced with cryosection and stained by hematoxylin and eosin for analysis.

***In vitro* screening of candidates for supra-adjuvant**

The effect of TLR 7/8a and IDO inhibitor in antigen-presenting cells was characterized based on released cytokines. RAW 264.7 cells (3×10^5 cell plate^{-1}) were cultured for 24 h in a

6-well plate in the presence of (a) R837 or R848 and (b) R848 (50 ng mL^{-1}) and an IDO inhibitor (1-MT, NLG919, or EPT). The supernatant was analyzed for the quantification of TNF-α and IL-10 using ELISA kits (BD Biosciences), according to the manufacturer's instructions.

IDO activity and expression analysis

IDO activity comparisons, between soluble EPT and soluble NLG919 and between AIMS(EPT) and soluble EPT, were performed using a HeLa assay. HeLa cells (5×10^3 cell $well^{-1}$) were cultured for 48 h in a 96-well plate in the presence of human IFN-γ (50 ng mL^{-1}) and the indicated samples. The supernatant was used to quantify kynurenine. 100 μL of supernatant was incubated with 50 mL of 30 % trichloroacetic acid (Sigma-Aldrich) for 40 min at 50 ℃, to remove all protein, followed by the centrifugation at 10,000 g for 10 min. A total of 100 mL of supernatant was mixed with 100 mL of Ehrlich's reagent (2 % 4-dimethylaminobenzaldehyde (Sigma Aldrich) in glacial acetic acid (Merck)) in a 96-well plate for 10 min. Absorbance was measured at 490 nm using a plate reader (VersaMax Microplate Reader, Molecular Devices).

IDO activity quantification in 4T1 tumor cells, BMDCs, and in vivo tissues (tumor and TDLN) was performed using an IDO1 activity assay kit (Abcam), according to the manufacturer's instructions. IDO expression was analyzed by western blot. Tissue (tumor and lymph node) excised from mice was lysed in RIPA buffer (Thermo Fisher Scientific) containing a protease inhibitor cocktail (Sigma Aldrich) and incubated on ice for 15 min. The sample was centrifuged at 14,000 × g for 15 min, and the supernatant was used for further analysis. The amount of protein in each sample was quantified with a BCA protein assay kit (Thermo Fisher Scientific). Equal amounts of protein mixed with SDS-PAGE loading buffer (Biosesang) were denatured at 96 °C for 5 min. After gel electrophoresis and protein transfer, the membrane was incubated with primary and secondary antibodies sequentially.

***In vitro* dendritic cell maturation analysis**

Immature BMDCs were generated from the bone marrow of BALB/c mice (female, 6-week-old, Orient Bio). Briefly, cells from the tibias and femurs were collected and red blood cells were depleted by red blood cell lysis buffer (BioLegend). Cells (2×10^6 cell plate^{-1}) were cultured in 100 mm × 25 mm Petri dishes (Corning) with complete RPMI 1640 medium containing 20 ng mL^{-1} GM-CSF. After 3 d, the cells were supplemented with fresh medium containing 20 ng mL^{-1} GM-CSF. After 7 d, differentiated cells were harvested, washed, and used for in vitro experiments.

4T1 cells (3×10^5 cell plate^{-1}) were treated with AIMS(PTX) (500 ng mL^{-1}) for 24 h in a 6-well plate and the supernatant was collected. BMDCs (1×10^6 cell plate^{-1}) were cultured for 24 h in a 6-well plate in the presence of AIMS, AIMS(EPT) (500 ng mL^{-1}), AIMS(R848) (500 ng mL^{-1}), supernatant from AIMS(PTX)-treated 4T1 tumor cells, or their combinations. The supernatant of the BMDCs was collected and analyzed for the quantification of IFN-γ, TNF-α and IL-10 using ELISA kits (BD Biosciences), according to the manufacturer's instructions. BMDCs were stained with FITC-conjugated anti-CD11c and APC-conjugated anti-CD40, and APC-conjugated anti-CD80 or APC-conjugated anti-CD86 antibodies for analysis by flow cytometry.

***In vitro* effect of AIMS(PTX)**

AIMS(PTX) induced the apoptosis of 4T1 cells. 4T1 cells (3×10^5 cells well^{-1}) in 6-well plates were cultured for 24 h in the presence of PTX, AIMS(PTX), AIMS(blank) and PBS. Apoptosis was analyzed by a FITC Annexin-V Apoptosis Detection Kit (BD Biosciences), according to the manufacturer's instructions, and a BD FACSCanto™ II flow cytometer.

ICD induced by AIMS(PTX) was characterized by the release of HMGB1 and exposure of Calreticulin (CRT). 4T1 cells (5×10^5 cells well^{-1}) were cultured for 24 h in FBS-free media,

followed by treatment with AIMS(PTX). The supernatant was analyzed for the presence of HMGB1 by an ELISA kit (Arigo Biolaboratories), according to the manufacturer's instructions. CRT exposure in 4T1 cells treated with PTX, AIMS(PTX), AIMS(blank) and PBS for 4 h was analyzed by a BD FACSCanto™ II flow cytometer. 4T1 cells were stained with primary and secondary antibodies for CRT detection, stained with Hoechst 33342 solution (1 mg mL^{-1}, Thermo Fisher Scientific) and fixed in 4 % (w/v) paraformaldehyde. Fluorescence images were obtained by a DeltaVision™ PD system (GE Life Sciences).

In vivo toxicity comparison of free drugs and AIMS(EPT, R848, PTX)

To compare the toxicity of free drugs and AIMS(EPT, R848, PTX) , BALB/c mice were subcutaneously injected with 5×10^5 4T1 tumor cells in the right flank and maintained for 5 d. After the average tumor volume reached 50 mm^3, control (PBS), solution containing free drug dissolved at 1, 1.5, and 3 times higher than AIMS(EPT, R848, PTX) , and AIMS(EPT, R848, PTX) were intratumorally injected 4 times at 2 d intervals. The amount of R848, EPT, and PTX used for injection were 25, 25, and 25 μg per injection for the 1-fold concentration group and AIMS(EPT, R848, PTX) group; 37.5, 30, and 37.5 μg per injection of each drug for the 1.5-fold concentration group; and 75, 60, and 75 μg per injection for the 3-fold concentration group. Tumor volume was measured with an electronic digital calliper (Mitutoyo, Japan), every 3 d from the first injection and calculated with the following formula: tumor volume(mm^3) = $0.5 \times$ length(mm) $\times$ (width (mm))^2. The body weight was measured with an electronic scale (WK-4C, CAS, Korea) during the same period.

In vivo cytokine analysis

To analyze the levels of released cytokines in the tumor or TDLN, tissue (tumor and lymph node) excised from mice was lysed in CelLytic MT cell Lysis Reagent (Sigma Aldrich) with protease inhibitor cocktail (Sigma Aldrich) and physically chopped into small pieces. The

lysate was centrifuged at 10,000 g for 10 min at 4 °C, and the supernatant was collected and analyzed for the quantification of IFN-γ, IL-12(p70), and IL-10 using ELISA kits (BD Biosciences) or TGF-β using an ELISA kit (Abcam), according to the manufacturer's instructions. The amount of protein in each sample was quantified with a BCA protein assay kit (Thermo Fisher Scientific).

***In vivo* immune cell profile analysis**

To analyze the immune cell profile of tumor or lymph node, the tissues excised from mice were immersed in DMEM and physically chopped into small pieces. The tissue was resuspended in DMEM containing 1 mg mL^{-1} collagenase D (Sigma Aldrich) and incubated for 40 min at 37 °C in a shaking incubator. The solution was filtered with a 70-mm cell strainer (Falcon) and washed twice with DPBS(1X) solution. After removing the supernatant of the washed cells, the cell staining antibody diluted in 1 % BSA solution was added and the cells were incubated at 4 °C in the dark for 30 min. Following the staining step, the cells were washed twice and fixed with 4 % (w/v) paraformaldehyde for 20 min at 25 °C. To characterize Foxp3, intracellular staining was conducted before the fixation step by using the True-Nuclear™ Transcription Factor Buffer Set (BioLegend, CA, USA), according to the manufacturer's instructions. Cells were analyzed by a FACS Canto™ II flow cytometer.

Evaluation of the *in vivo* adjuvant effect in a subcutaneous tumor model

To evaluate the therapeutic effect of supra-adjuvant-loaded AIMS, BALB/c mice were subcutaneously injected with 5×10^5 4T1 tumor cells in the right flank and maintained for 5 d. After the average tumor volume reached 50 mm^3, control (PBS), AIMS (Blank), AIMS (EPT), AIMS (R848), and AIMS (EPT, R848) were intratumorally injected four times at 2 d intervals. The amount of drug injected was 25 μg R848 per injection and 25 μg EPT per injection. The tumor volume was measured with an electronic digital calliper every 3 d. The primary tumor

and tumor-draining lymph node were collected for tumor weight measurement, immune cell profiling, and cytokine analysis, 3 d after the last injection.

***In vivo* tumor challenge in a distant tumor**

To demonstrate the systemic therapeutic effect of AIMS(EPT, R848, PTX) , BALB/c mice were subcutaneously inoculated with 5×10^5 4T1 tumor cells in the right flank and maintained for 5 d. After the average tumor volume reached 50 mm^3, control (PBS), AIMS(EPT, R848, PTX) , and lyophilized AIMS(EPT, R848, PTX) were intratumorally injected four times at 2 d intervals. The amounts of drug amount injected were 25 μg R848 per injection, 25 μg EPT per injection, and 25 μg PTX per injection. One day after the first injection, 5×10^5 4T1 tumor cells were inoculated in the opposite flank. The tumors were excised from the mice for weight measurement and immune cell profile analysis, 3 d after the last injection.

***In vivo* tumor challenge with an immune checkpoint blockade**

To verify the anti-tumor therapeutic efficacy of AIMS(EPT, R848, PTX) with an immune checkpoint blockade, BALB/c mice were subcutaneously inoculated with 5×10^5 4T1 tumor cells in the right flank and maintained for 5 d. After the average tumor volume reached to 50 mm^3, control (PBS), α-PD-1, α -PD-L1, AIMS(EPT, R848, PTX) , α -PD-1 + AIMS(EPT, R848, PTX) , and α -PD-L1 + AIMS(EPT, R848, PTX) were injected four times at 2 d intervals. AIMS(EPT, R848, PTX) was intratumorally injected, and immune checkpoint blockades were intraperitoneally injected separately. The amounts of drug injected were 25 μg R848 per injection, 25 μg EPT per injection, 25 μg PTX per injection, and 100 μg immune checkpoint blockade per injection. The tumor volume was measured with an electronic digital calliper every 3 d from the first injection, and the survival test was conducted for 50 d.

Randomly selected mice from each group were sacrificed for tumor weight measurement and immune cell profile analysis 3 d after the last injection.

Lung metastasis

To prove the lung metastasis inhibiting effect of AIMS(EPT, R848, PTX) , BALB/c mice were subcutaneously inoculated with 5×10^5 4T1 tumor cells in the right flank and maintained for 5 d. After the average tumor volume reached 50 mm^3, control (PBS), AIMS(EPT, R848, PTX) , and lyophilized AIMS(EPT, R848, PTX) were intratumorally injected four times at 2 d intervals. The amounts of drug injected were 25 μg R848 per injection, 25 μg EPT per injection, and 25 μg PTX per injection. Thirty days after tumor inoculation, the mice were euthanized, and the lungs of the mice were stained with India ink. Then, the lungs were separated from the mice and submerged in Fekete's solution (100 mL of 70 % ethanol, 10 mL of 4 % formaldehyde, and 5 mL of 100 % glacial acetic acid) for decolorization of the metastatic nodules. The number of metastatic nodules were counted 1 d after immersion.

Statistical analysis

All results are presented as the means ± standard deviations. Statistical analysis was conducted by one-way ANOVA with corresponding Tukey's multiple comparison for multiple groups of data, two-tailed unpaired Student's t test for comparisons between two groups, and the Kaplan–Meier method for survival tests. Significance was indicated by $P < 0.05$ (*P < 0.05; **P < 0.01; ***P < 0.005; ****P < 0.001). GraphPad Prism 8 (GraphPad software) and Excel 2016 (Microsoft Corporation) were used for all statistical analyzes.

	Cargo	Base solvent	Co-surfactant	Size (nm)	PDI	Adjuvant concentration	Encapsulation efficiency
**AIMS(Blank)**	-	-	-	233.5 ± 45.3	0.213 ± 0.018	-	-
**AIMS(R837)**	Imiquimod	Chloroform	Oleic acid	183.6 ± 38.7	0.234 ± 0.020	1.25 mg mL^{-1}	87.6%
**AIMS(R848)**	Resiquimod	Chloroform	Oleic acid	181.7 ± 36.5	0.226 ± 0.017	1.25 mg mL^{-1}	51.3%
**AIMS(EPT)**	Epacadostat	Ethyl alcohol	-	137.0 ± 34.5	0.249 ± 0.023	2.00 mg mL^{-1}	97.7%
**AIMS(1-MT)**	1-Methyl-D-tryptophan	Ethyl alcohol	Oleic acid	159.5 ± 46.7	0.238 ± 0.021	2.00 mg mL^{-1}	36.9%
**AIMS(NLG)**	NLG919	Ethyl alcohol	Oleic acid	184.3 ± 45.4	0.274 ± 0.026	2.00 mg mL^{-1}	78.9%
**AIMS(PTX)**	Paclitaxel	Chloroform	Oleic acid	151.6 ± 35.4	0.215 ± 0.014	1.25 mg mL^{-1}	95.4%
**AIMS(GEM)**	Gemcitabine	Ethyl alcohol	Oleic acid	148.9 ± 34.1	0.253 ± 0.024	1.25 mg mL^{-1}	12.8%

Table S1. AIMS platform. Squalene based nano-size emulsion platform "AIMS" can contain different types of immune modulating components. The immune modulating cargo was encapsulated in oil phase by solvent-evaporation method with base solvent and co-surfactant. The size of emulsion was characterized by number distribution of dynamic light scattering (DLS) measurement, and the encapsulation efficiency was characterized the amount of component in emulsion.

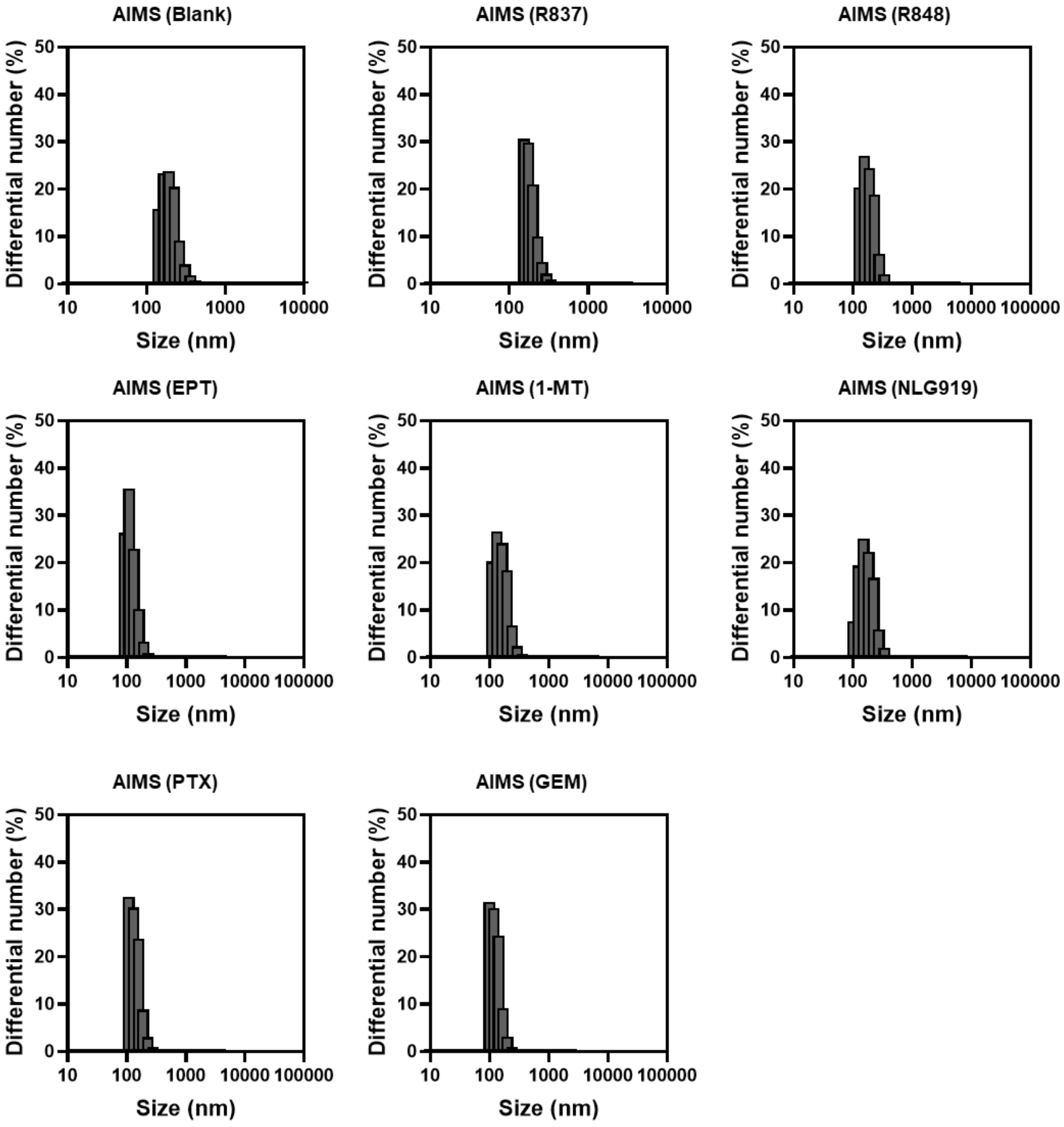

Figure S1. Size distribution of AIMS platform. AIMS synthesized with each contained drug purified by centrifuge filter and the size distribution was characterized by the DLS.

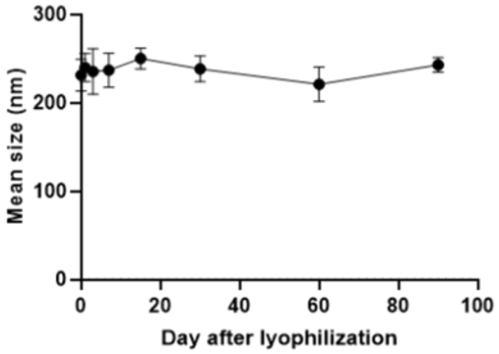

Figure S2. Stability of AIMS system. Size distribution of AIMS after synthesizing in room temperature for long period (n = 3).

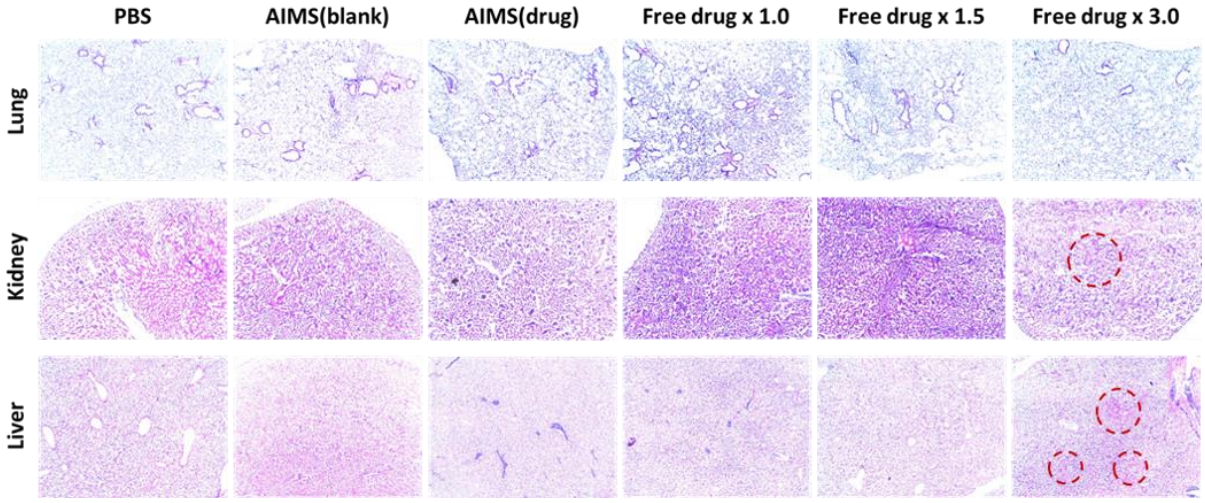

Figure S3. H&E staining of different organs after AIMS injection. Lung, kidney, and liver of each group was stained after 4 times injection with 3 days interval. Drug means the combination of 25 μg of R848, 25 μg of epacadostat (EPT), and 25 μg of paclitaxel (PTX). The red circles indicate a cell distortion site.

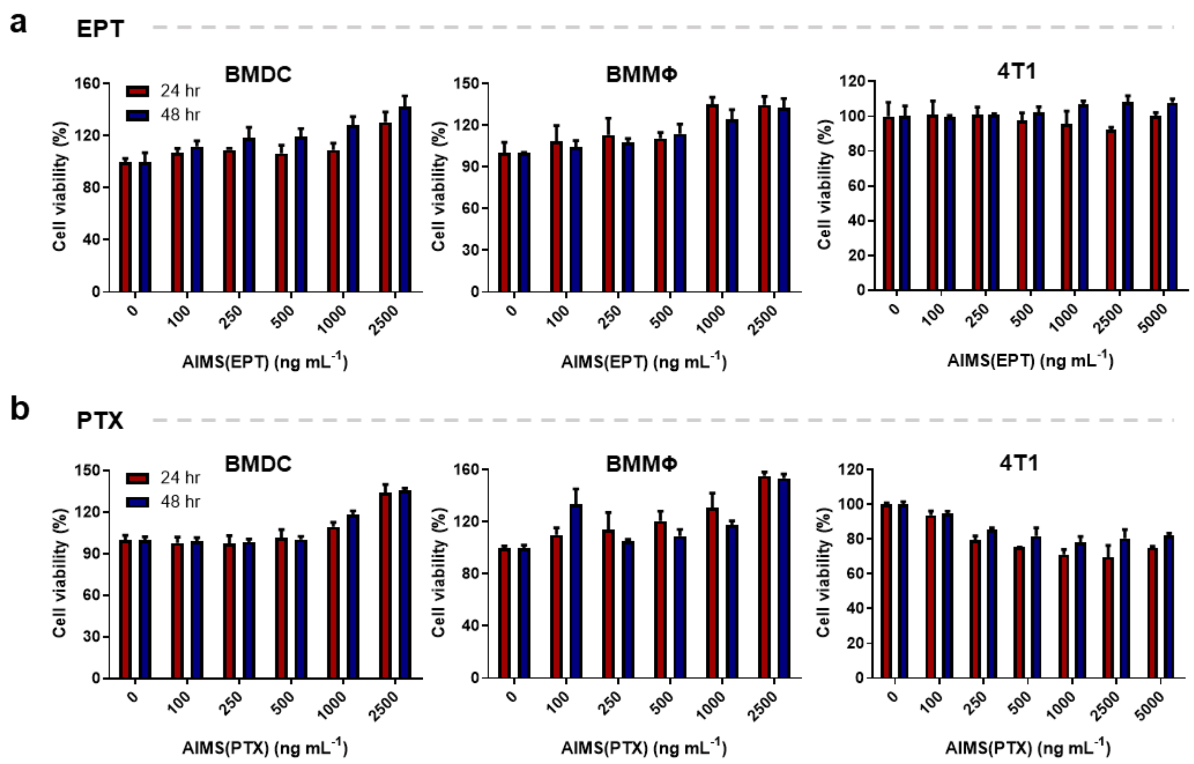

Figure S4. Effects on immune cell and tumor cell of IDO inhibitor and PTX. *In vitro* cell proliferation assay about different two immune modulating components **(A)** EPT and **(B)** PTX was conducted by MTT assay at bone-marrow derived dendritic cells (BMDC), bone-marrow derived macrophage (BMMQ), and 4T1 tumor cells.

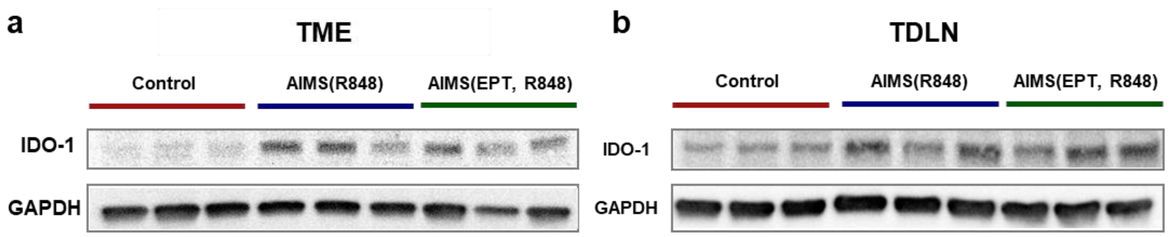

Figure S5. IDO-1 expression induced by R848. Western blot analysis of IDO-1 expression in a) tumor microenvironment (TME) and b) tumor draining lymph node (TDLN).

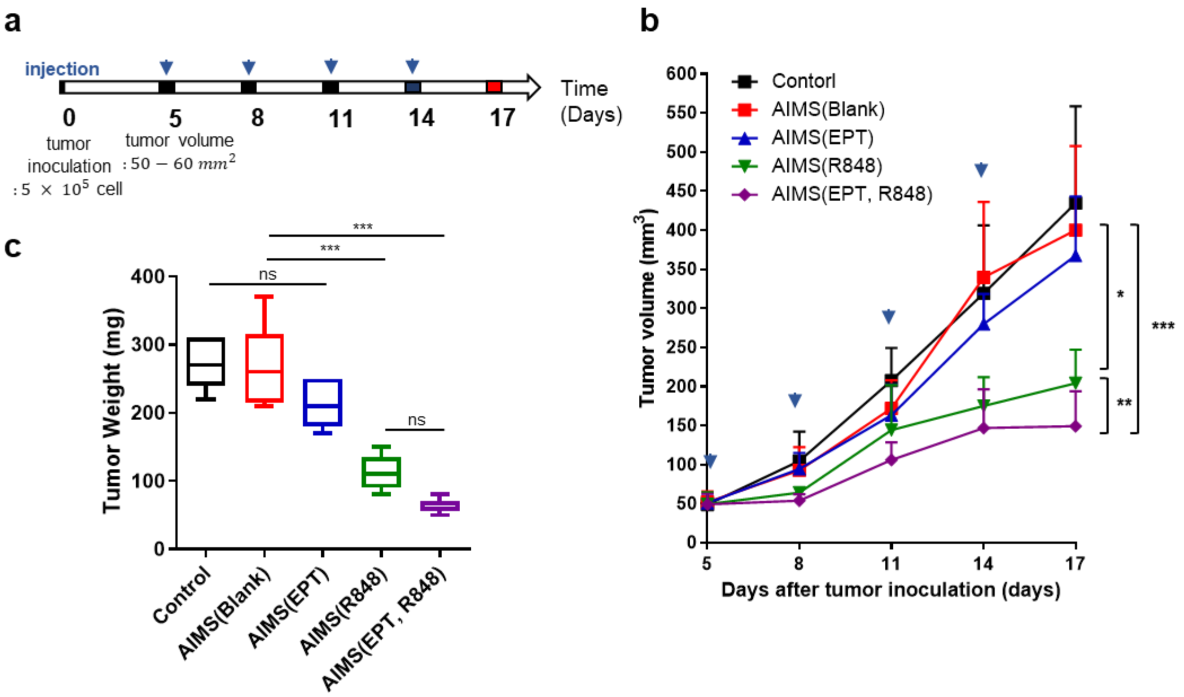

Figure S6. Anti-tumor effect of adjuvant system (TLR 7/8 agonist & IDO inhibitor). Mice were treated four times with three-day intervals when the tumor reached 50-60 mm^3 in size. Control (PBS), AIMS(blank), AIMS(EPT), AIMS(R848), and AIMS(EPT, R848). The *in vivo* experiment was followed by **(A)** mice experiment schedule. **(B)** Tumor growth curve (n = 6) and **(C)** tumor weight curve (n = 5) was measured. Data are presented as the mean ± SD. P values were determined by one-way ANOVA (*$P < 0.05$, **$P < 0.01$, ***$P < 0.005$; ns, not significant).

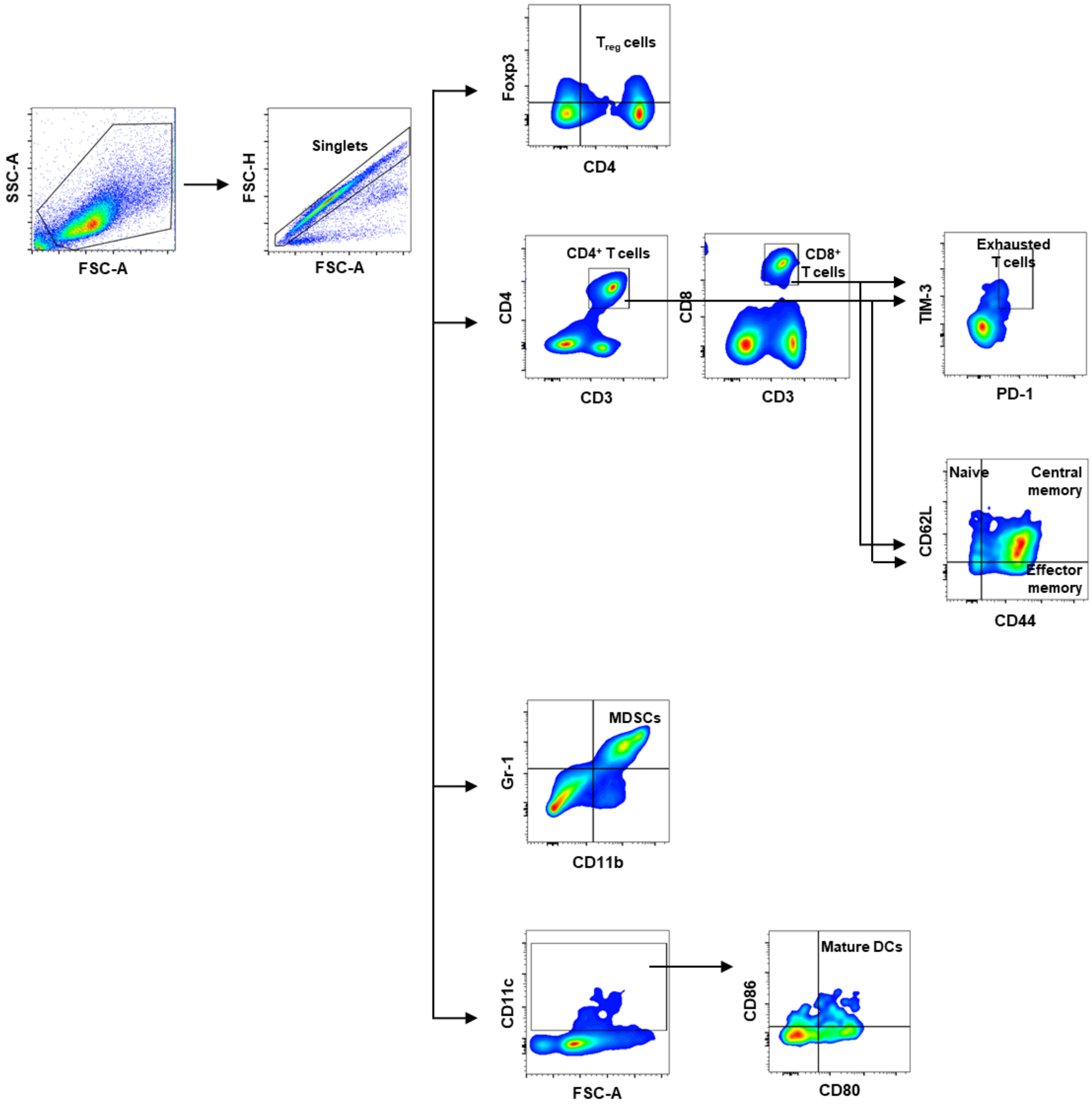

Figure S7. Gating strategy for flow cytometric analysis of immune cell populations. Initial population was gated to remove the cell debris, dead cells (small FSC and SSC), and only live population remained. Second population was gated to select the singlets. **(A)** Treg ($CD4^+Foxp3^+$) cells presented on Figure 6g, 6j. **(B)** $CD3^+CD4^+$, $CD3^+CD8^+$ T cells presented on Figure 6f, 6i, 7f, 7h, S17. **(C)** Exhausted T cells presented on Figure 6k. **(D)** Memory T cells presented on Figure S12. **(E)** MDSCs ($CD11b^+Gr\text{-}1^+$) presented on Figure 6h. **(F)** Mature DCs ($CD80^+CD86^+$) presented on Figure S8.

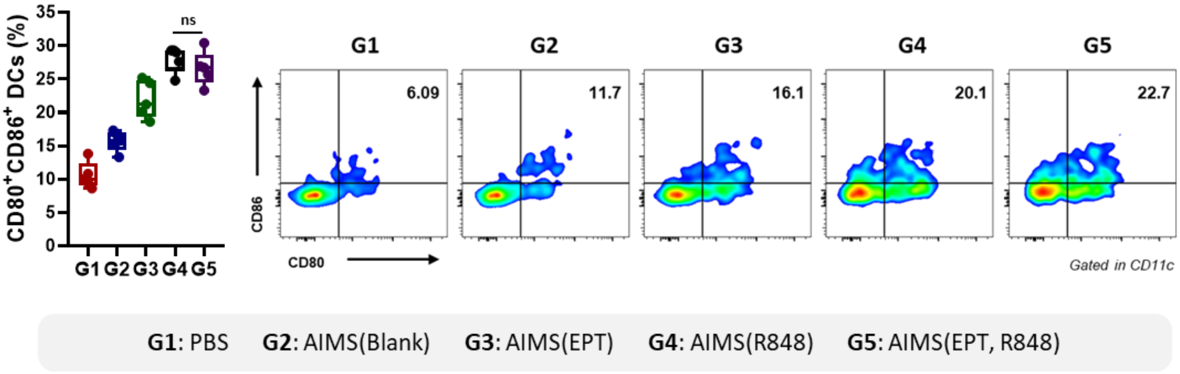

Figure S8. Flow cytometric analysis of supra-adjuvant-mediated DC maturation. Flow cytometric analysis of $CD80^{+}CD86^{+}$ DCs and representative flow cytometric dot plots.

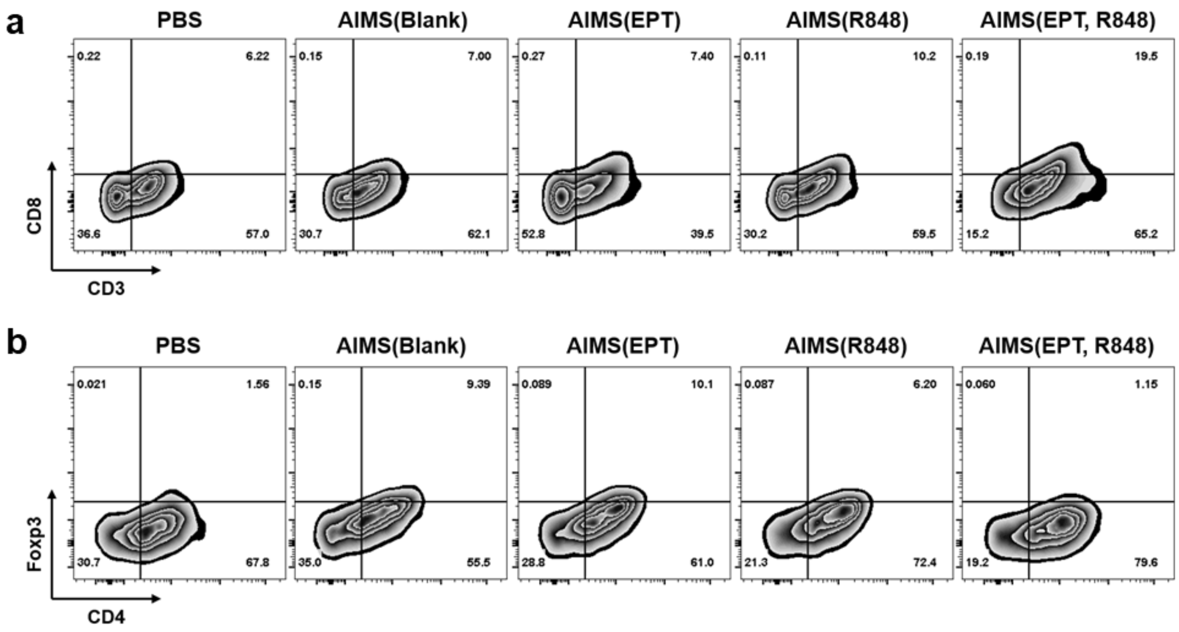

Figure S9. Representative flow cytometric dot plot of supra-adjuvant-mediated immune modulation in the TME. Representative gating of **(A)** $CD8^+$ T-cells ($CD3^+CD8^+$), and **(B)** Treg cells ($CD4^+FOXP3^+$) in TME.

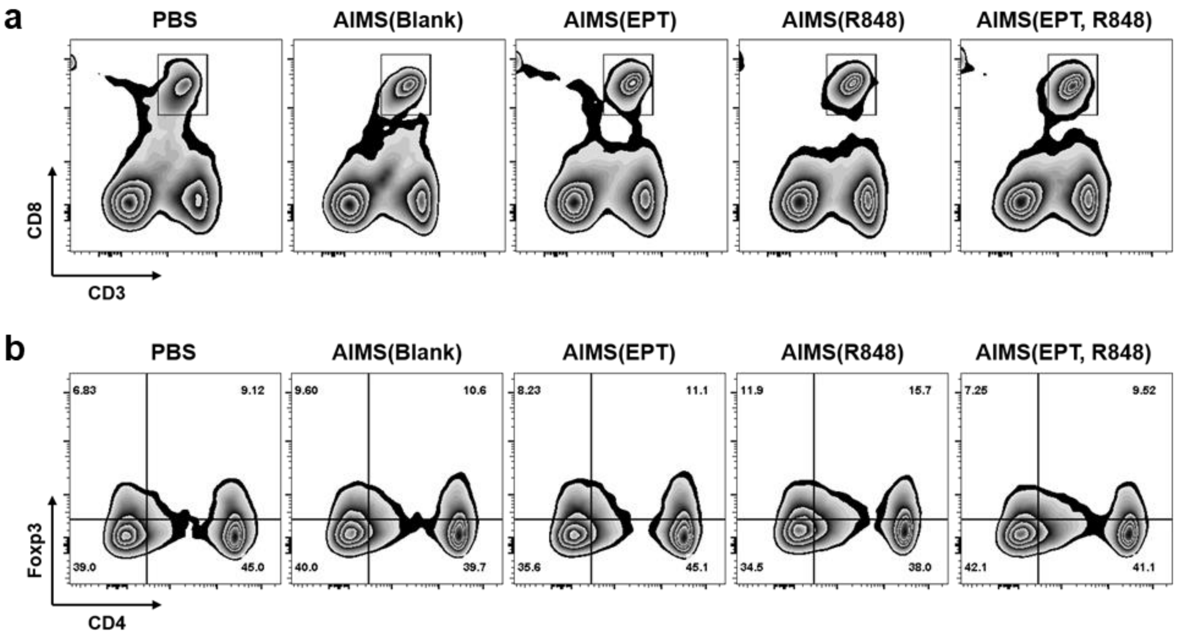

Figure S10. Representative flow cytometric dot plot of supra-adjuvant-mediated immune modulation in the TDLN. Representative gating of **(A)** $CD8^+$ T-cells ($CD3^+CD8^+$), and **(B)** Treg cells ($CD4^+FOXP3^+$) in TDLN.

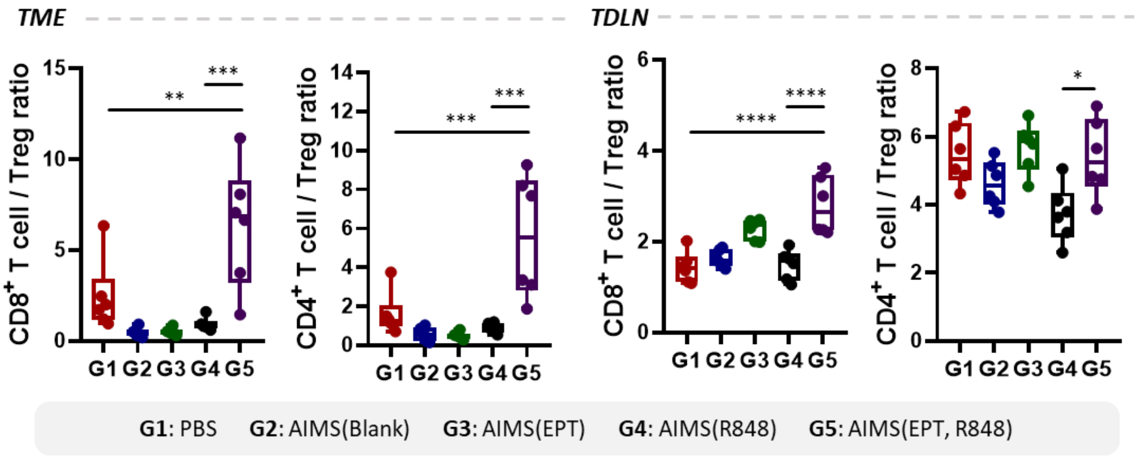

Figure S11. Effector T cell – Regulatory T cell ratio in TME and TDLN. Mice were treated four times with three-day intervals when the tumor reached 50-60 mm^3 in size. Flow cytometry analysis was conducted for the ratio of $CD8^+$ T cell and Treg cell in the TME and the TDLN. Data are presented as the mean ± SD. P values were determined by one-way ANOVA (*P < 0.05, ***P < 0.005, ****P < 0.001).

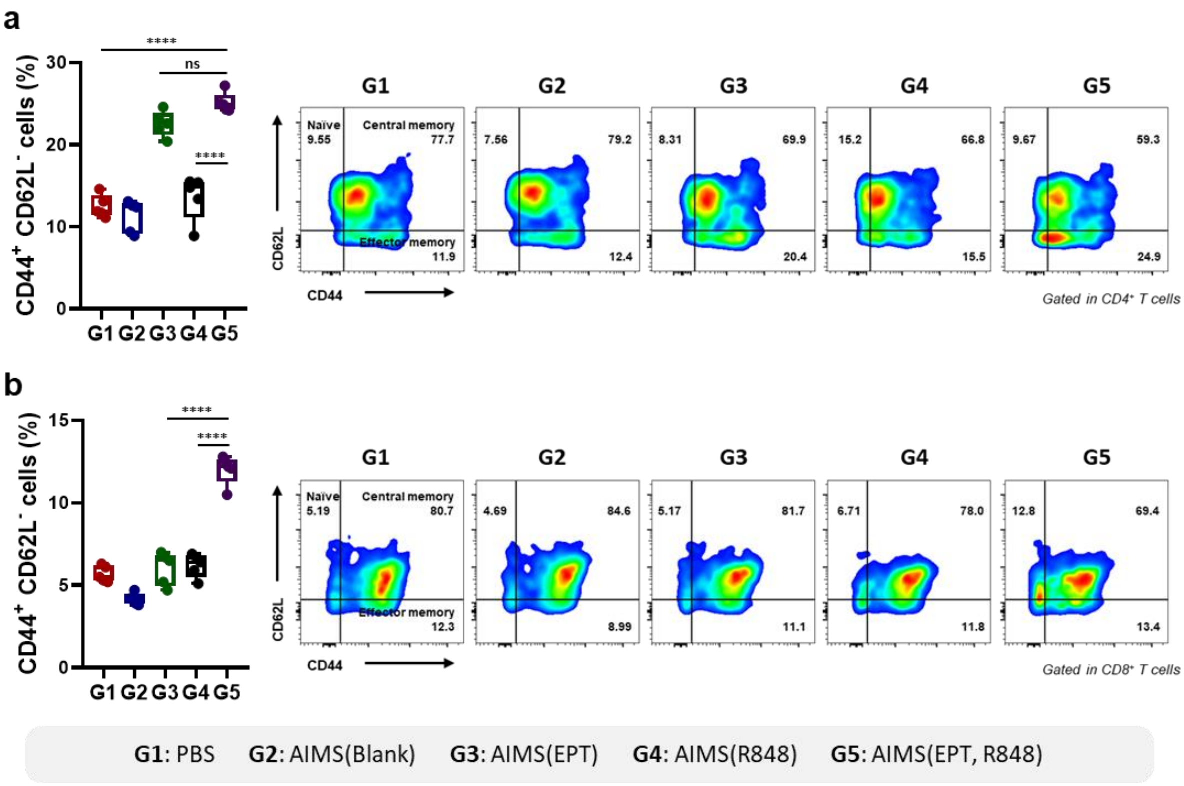

Figure S12. Flow cytometric analysis of supra-adjuvant-mediated effector memory T cell generation. Flow cytometric analysis of **(A)** $CD44^+CD62L^-$ T cells in $CD4^+$ T cells and their representative flow cytometric dot plots and **(B)** $CD44^+CD62L^-$ T cells in $CD8^+$ T cells and their representative flow cytometric dot plots in LN.

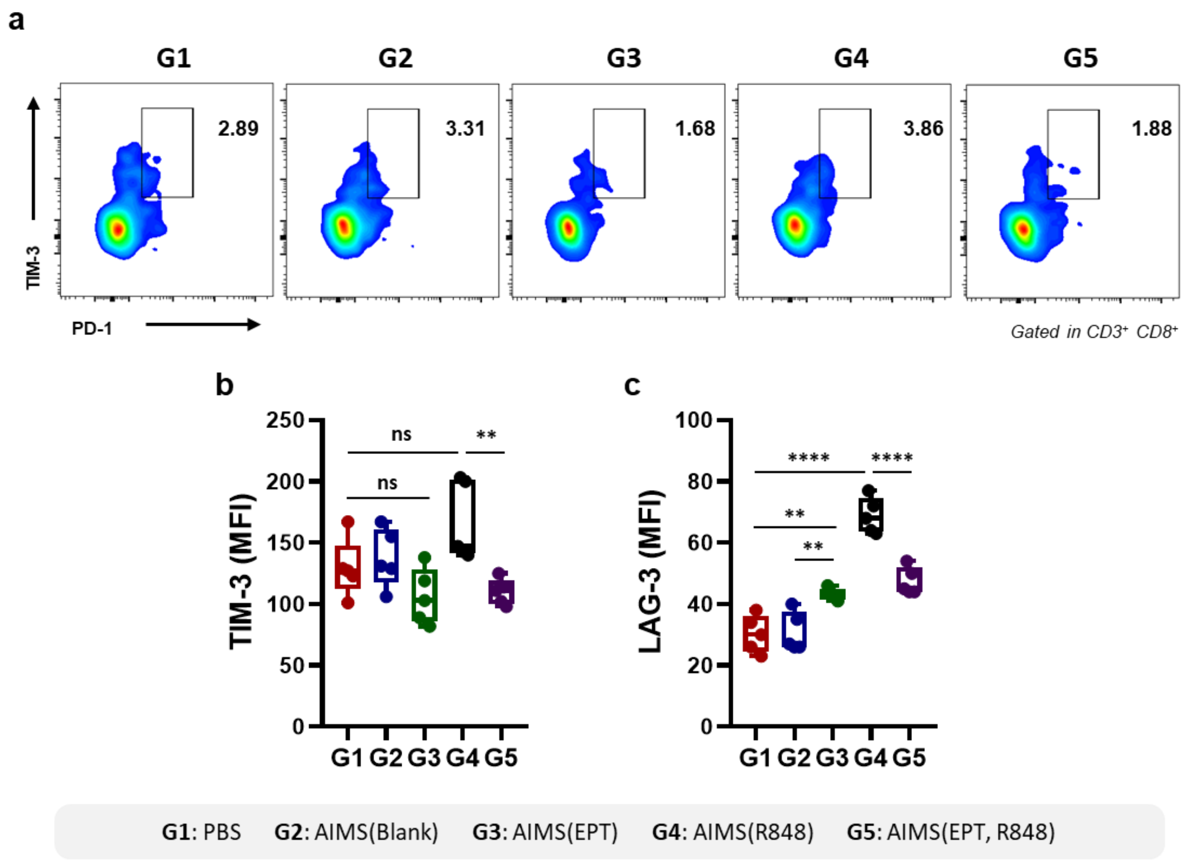

Figure S13. Flow cytometric analysis of supra-adjuvant-mediated exhaustion blocked of T cells. (A) Representative flow cytometric dot plot gating of PD-1$^+$TIM-3$^+$ exhausted T cells **(B)** MFI value of exhaustion marker TIM-3 in CD8$^+$ T cells, and **(C)** MFI value of exhaustion marker LAG-3 in CD8$^+$ T cells.

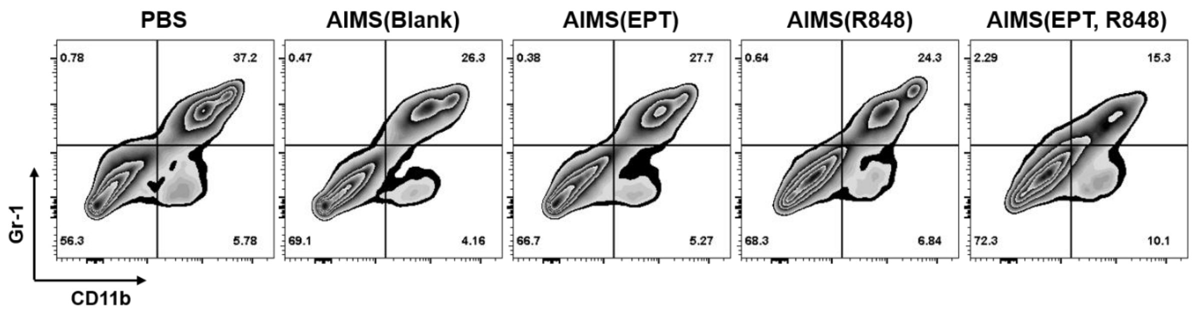

Figure S14. Representative flow cytometric dot plot of supra-adjuvant-mediated immune modulation in TME. Representative gating of MDSC ($CD11b^{+}Gr\text{-}1^{+}$) in TME.

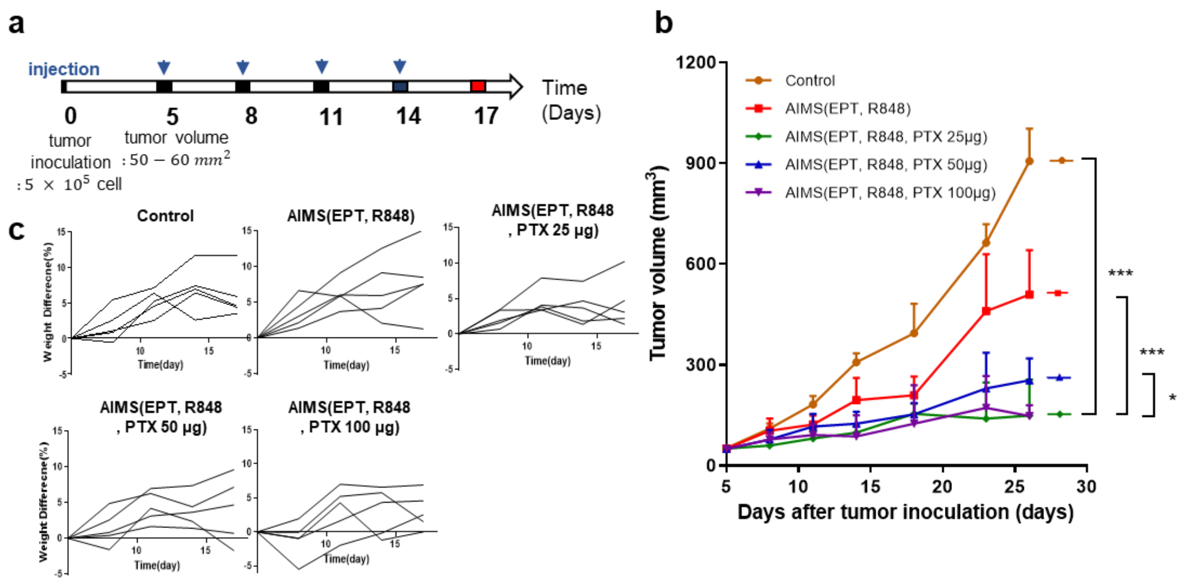

Figure S15. AIMS(PTX) dose determination test. Mice were treated four times with three-day intervals when the tumor reached 50-60 mm^3 in size. Control (PBS), AIMS(EPT, R848), AIMS(EPT, R848 and PTX 25 μg), AIMS(EPT, R848 and PTX 50 μg), and AIMS(EPT, R848 and PTX 100 μg). The *in vivo* experiment followed **(A)** mice experiment schedule. **(B)** Tumor growth curve (n = 5) and **(C)** body weight curve (n = 5) was calculated from the experiment. Data are presented as the mean ± SD. P values were determined by Student's t test (*P < 0.05, ***P < 0.005).

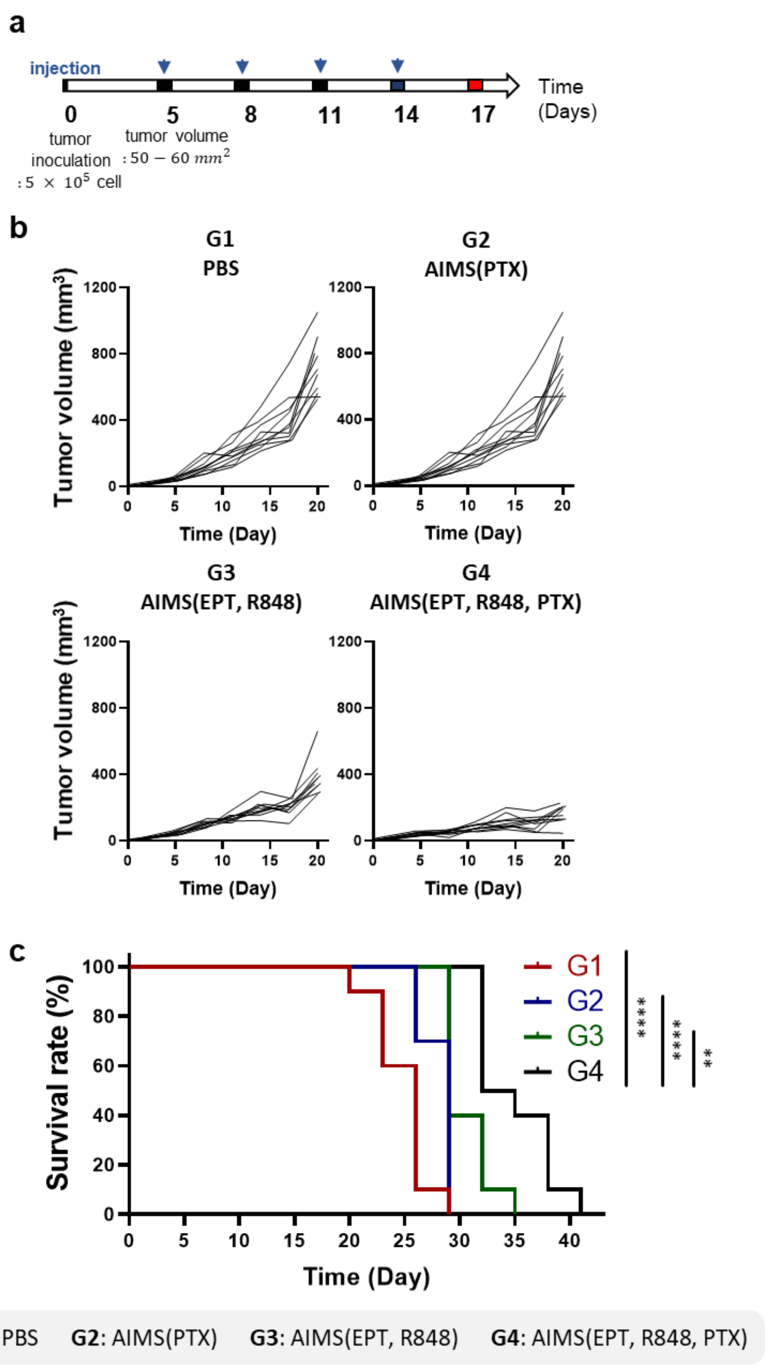

Figure S16. Antitumor effect (tumor growth, survival rate) of the combination of supra-adjuvant and PTX. Mice were treated as follow. G1, PBS; G2, AIMS(PTX); G3, AIMS(EPT, R848); G4, AIMS(EPT, R848, PTX). The sample was started to treat 4 times, 5 days after tumor inoculation with 3 days interval following **(A)** mice experiment schedule. **(B)** The tumor growth curve (n = 10) and **(C)** survival rate (n = 10) was calculated from the experiment. P values were determined by Log-rank test (**$P < 0.01$, ****$P < 0.005$).

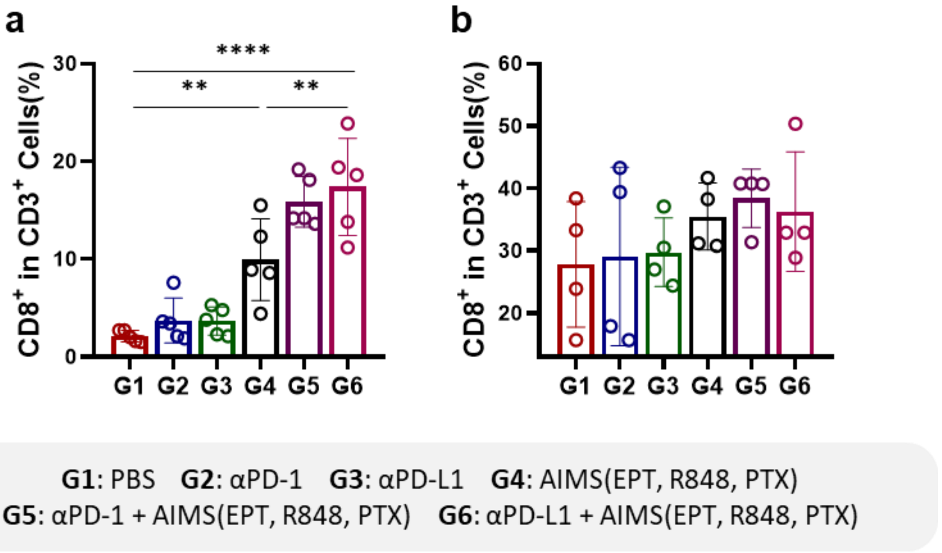

Figure S17. $CD8^+$ T cell analysis for ICBT combination with AIMS platform. 4T1 tumor or TC1 tumor bearing mice were treated with indicated samples 4 times with 3 days interval starting at 5 days after tumor inoculation. Flow cytometry analysis of $CD8^+$ T cells in **(A)** 4T1 tumor model (n = 5) and **(B)** TC1 tumor model (n = 4). Data are presented as the mean ± SD. P values were determined by one-way ANOVA (***P* < 0.01, *****P* < 0.001).

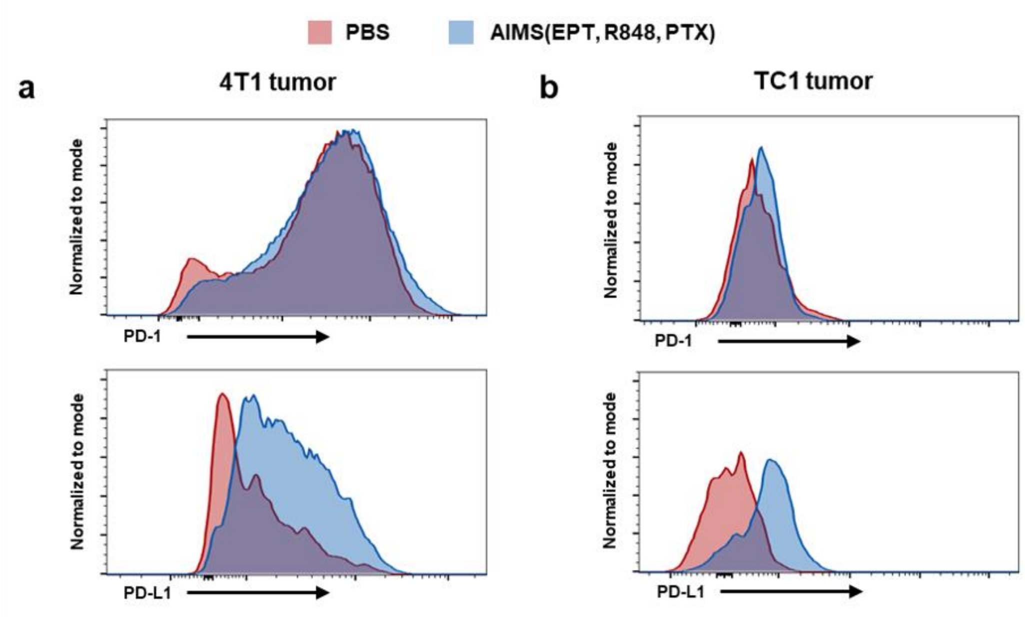

Figure S18. Representative flow cytometric histogram of PD-1 and PD-L1 in the TME. Representative histogram of PD-1 in T cells ($CD3^+$ cells) and PD-L1 in non-lymphocyte cells ($CD45^-$ cells) analyzed in TME of **(A)** 4T1 tumor model and **(B)** TC1 tumor model.

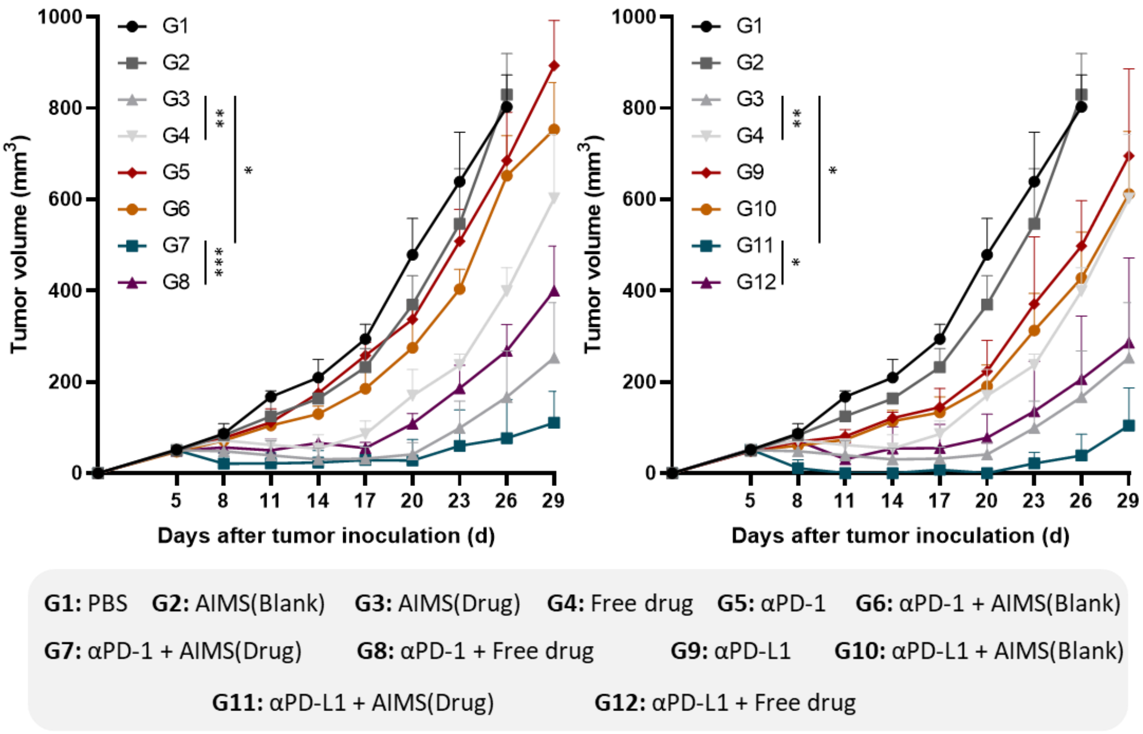

Figure S19. Enhanced antitumor response by AIMS platform combination with immune-checkpoint inhibitor αPD-1 and αPD-L1 in 4T1 tumor. Tumor growth curve when AIMS platform injected with **(A)** αPD-1 (n = 6) and **(B)** αPD-L1 (n = 6). Drug means the combination of 25 μg of R848, 25 μg of EPT, and 25 μg of PTX. Each sample was treated four times, 5 d after tumor inoculation at 3 d intervals, and the tumor growth was calculated. Data are presented as the mean ± SD. P values were determined by Student's t test at the endpoint (*$P < 0.05$, **$P < 0.01$, ***$P < 0.005$; ns, not significant).
